# Yuhuangmiao: the socio-cultural dynamics of a community between the steppes and the Chinese plains

**DOI:** 10.1007/s41826-024-00098-4

**Published:** 2024-12-09

**Authors:** Limin Huan, Ursula Brosseder

**Affiliations:** Department Prehistory, LEIZA (Leibniz-Zentrum Für Archäologie), Mainz, Germany

**Keywords:** Yuhuangmiao, Agro-pastoral, Northeast China, Chronology, Seriation, Correspondence analysis

## Abstract

The Yuhuangmiao culture emerged around the 7th to the 4th centuries BCE in northeastern China near Beijing. The burial ritual with stone layers, numerous animal deposits, and the material culture indicate a strong steppe connection. It is often used to support the narratives in the Chinese historical texts that people living in the area had a distinctive lifestyle from those in the southern, agricultural-based communities and that the two groups often had a hostile relationship. Based on the cemetery of Yuhuangmiao, we focus in our case study on the socio-cultural dynamics over time in communities of a small region between the steppes and the Chinese Central Plains and in confrontation with very different neighbors to the south and the northwest. We use a seriation and correspondence-based approach to propose a new chronology for three sites. The duration of the sites is divided into three phases, with a total span of around 200 years between 600 and 400 BCE. Our results show that the changes in the burial ritual and material culture reflect a community’s internal development and external communication with other regions. Based on our chronology, the elite members of the Yuhuangmiao community used objects with steppe designs and horses to express their identities and social power. This practice was most prominent in the first phase and waned over time. We suggest reconsidering previous interpretations of these practices as a decline of the Yuhuangmiao people and view it instead as an expression of intra-community changes, possibly as an indicator of a successful integration of a community with foreign immigrants in an area located between two very different systems: of the steppes and the Central Plains.

**Supplementary Information:**

The online version contains supplementary material available at 10.1007/s41826-024-00098-4.

## Introduction

The interaction between agricultural and pastoral groups is a central theme in world archaeology. In the first millennium BCE, agrarian communities occupied the lowland alluvial plains and the adjacent river valleys in today’s eastern China, which in the paper we call the Central Plains (*zhongyuan*).^1^ At the same time, people living between the steppes and the Central Plains often developed a more hybrid culture and lifestyle (Yang et al. 2020). The area of present-day China that stretches along its northern border is an intermediate region between steppes and deserts on the one side and alluvial plains on the other side. Historically, this region has always served as a connector, and its importance has been recognized and studied over the past decades, coined as the Arc by Jessica Rawson (Rawson 2017; 2023, p. 394; Rawson et al. 2021; other scholars use “northern cultural zone” or “Inner Asian frontier” to refer to different, but similar regions: Lin 2017; Linduff et al. 2018; Yang 2004). One striking aspect in this wider area is the emergence of a great number of local phenomena or groups that show distinct funerary practices and assemblages of artifacts, reflecting strong ties to the steppes as well as to the Central Plains (Lin 2017; Linduff et al. 2018; Wu’en 2007; Yang 2004). They are often called archaeological cultures. However, instead of considering them as discrete and essential groups or entities, we prefer to see these phenomena as more fluid, steering away from essentialistic groupings of people whose identities need to be discussed (Roberts and Linden 2011). After all, what unites these local phenomena is their local processing and fashioning in an area with connections to the steppes and the Central Plains.

**Fig. 1:**
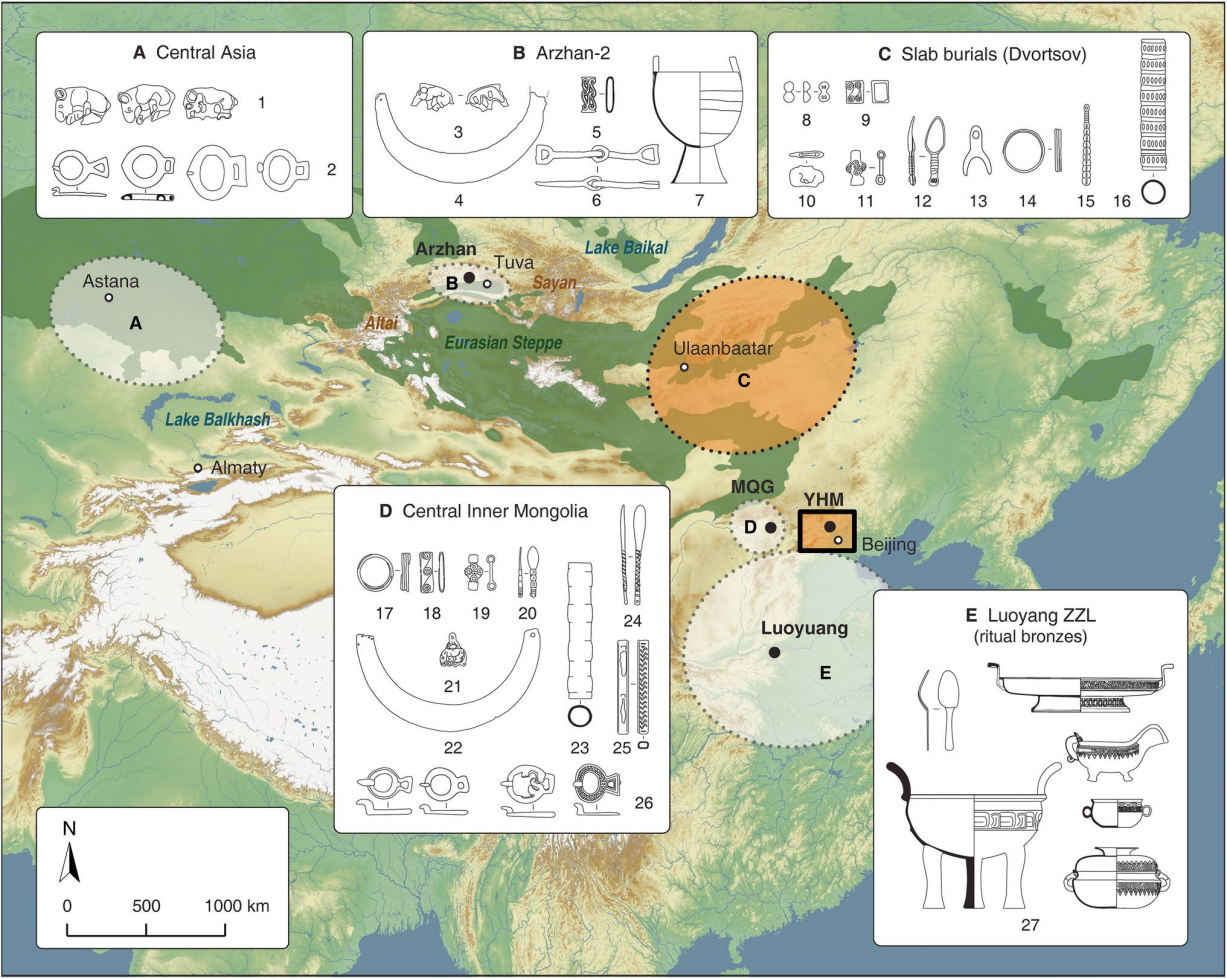
Regions and sites in East and Central Asia which are related to the Yuhuangmiao culture (the highlighted area), first millennium BCE. Regions: A: central and eastern Kazakhstan; B: Tuva; C: Transbaikal and eastern Mongolia; D: central Inner Mongolia; E: the Central Plains of China. Grave construction and burial rites in the Yuhuangmiao culture were closely related to those in the slab burials and the Dvortsov culture in Mongolia and Transbaikal (the orange color). Meanwhile, material cultures show that features in the Yuhuangmiao objects had three possible external sources: Central Asia, Mongolia, and the Central Plains. The existence of similar objects in central Inner Mongolia suggests that the region was also involved in the cultural interaction (objects modified from: Central Asia: Bernshtam 1952; Margulan et al. 1966; Roberts 2021; Rudenko 1960; Arzhan-2: Chugunov et al. 2010; slab burials and Dvortsov: Grishin 1981; Kirillov and Kirillov 1985; Kirillov 1988b; Tsybiktarov 1998; central Inner Mongolia: Cao 2006; Cao et al. 2009; Liu 1991; Neimenggu 1986; Luoyang: Zhongguo 1959; Eurasian steppe: after Dinerstein et al. 2017; map by L. Huan and K. Hölzl)

In this paper, we focus on one such group in a basin of the Jundu Mountains, which are the western extension of the Yan Mountains, northwest of Beijing (Beijingshi 2007, p. 9; Linduff et al. 2018, pp.75–76; Rawson 2023, p. 179; Bunker and Brosseder 2022) (Fig. 1). In a small part of the Jundu Mountains there is the Yanhuai Basin, covered nowadays by the Guanting Reservoir (500 m asl) that collects the water of the Sanggan and Yang rivers. The local landscape combines forest-covered mountain slopes and river floodplains. This ecological mosaic facilitates diverse subsistence economies in the historical eras and at present, including farming, herding, and hunting (Chen 1938; Chen et al. 2022; Han 2010).

**Fig. 2:**
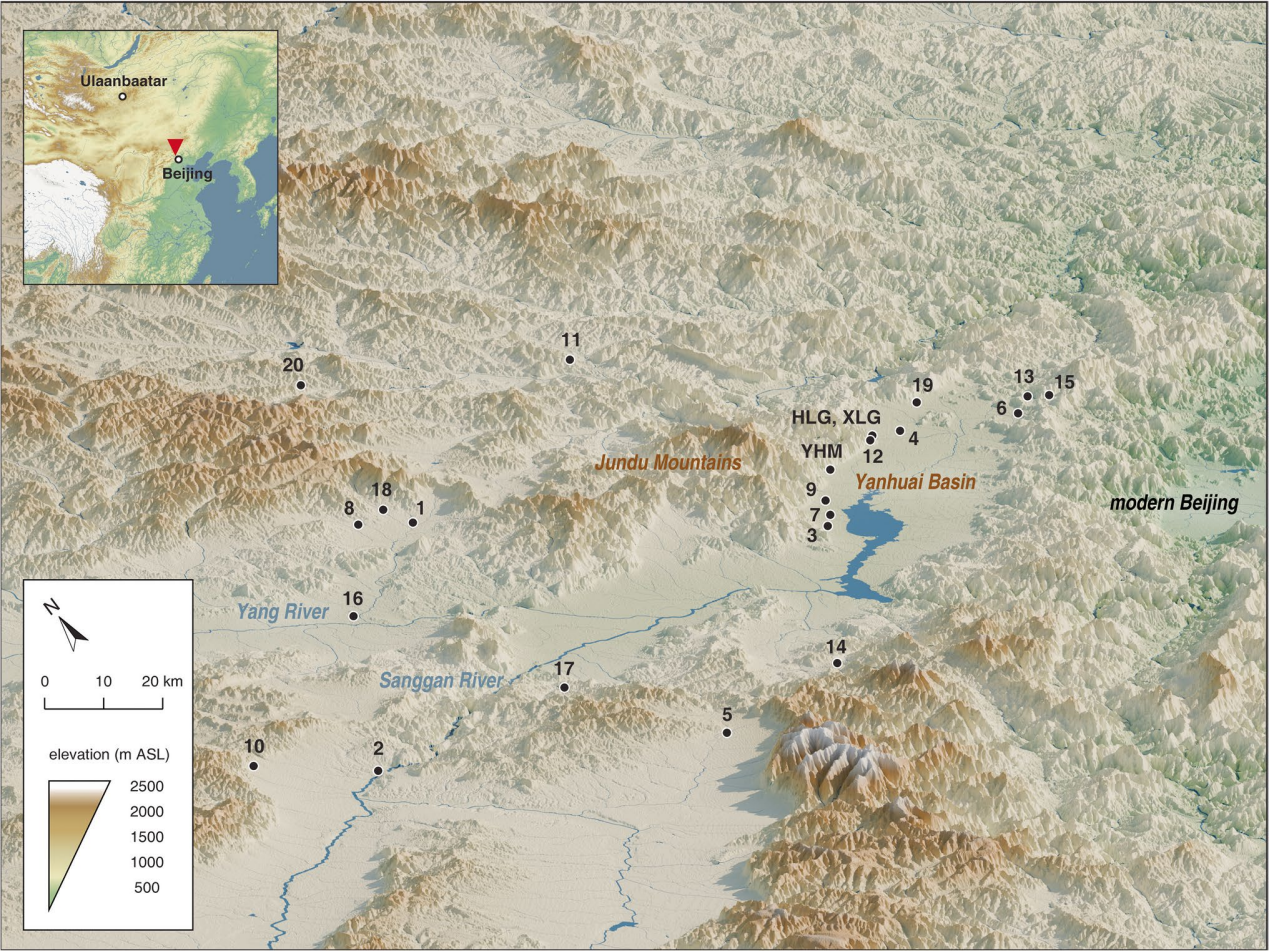
Sites around the Yanhuai Basin with objects associated with the Yuhuangmiao culture. The three major sites are marked on the map. Other sites: 1: Baimiao; 2: Banjing; 3: Beixinbao; 4: Changliying; 5: Daolazui; 6: Donghuiling; 7: Ganzibao; 8: Huangtupo; 9: Hujiaying; 10: Jiugou; 11: Liuchanggou; 12: Longqingxia; 13: Luojiatai; 14: Luopandi; 15: Matiwan; 16: Nihezi; 17: Sunjiagou; 18: Xiaobaiyang; 19: Xiaobao; 20: Zhongsuo (data: Online Resource 1; map by L. Huan and K. Hölzl)

Stray finds and material assemblages with objects, such as bronze daggers with straight blade edges and a straight handle, have often been found around the Yanhuai Basin. Numerous similar artifacts, with or without clear provenances, have long been collected in China and overseas (Bunker 1997; Bunker and Brosseder 2022; Bunker et al. 2002; Linduff 1997; So and Bunker 1995). Since the 1980s, around 20 cemeteries with the same material culture have been discovered and excavated (Jin 1989) (Fig. 2). The excavators first named the phenomenon the “Mountain Rong culture” and later renamed it to the Yuhuangmiao culture, after the eponymous site of Yuhuangmiao (YHM, 40°30′50.0″ N, 115°53′33.5″ E) (Beijingshi 2007; Hong 2022; 2014; Jin 1989; Yang 2003; “Rong” is a name from Chinese historical records, see later discussion). Two other excavated and well-published cemetery sites are Hulugou (HLG) and Xiliangguang (XLG) (Beijingshi 2010). All three sites are located on terraces (500–600 m asl) between the mountain slopes and the Guanting Reservoir. The excavation reports were published in 2007–2010, covering 400 graves from YHM, 153 from HLG, and 41 from XLG (Beijingshi 2007, 2010). The publications remain the most important source for understanding this regional group. Sites with similar objects but out of the Yanhuai Basin have also been associated with the Yuhuangmiao culture by some scholars (Online Resource 1).

Since the discoveries of these sites, scholars have noticed the remarkably extensive connections between the Yanhuai Basin and the steppes, especially regarding the forms of burials and artifacts (Lin 2017; Quan and Yang 2021; Shao and Yang 2022). Within the agro-pastoral intermediate zone of northern China, the group of Yuhuangmiao holds a special place as it is the only area where strong ties to the Arzhan-2 burial from Tuva become visible in the material culture. Even more astoundingly, in further north, Central Mongolia, such ties to Arzhan or more general the Early Iron Age in Tuva, were by and large absent. The most sensible and recent study on the importance of Yuhuangmiao, especially recognizing the role of the large tombs, has been presented by Rawson, who points to the steppe connection the tombs display, especially the analogous artifacts that connect the owners of some large tombs with the Arzhan-2 burial (Rawson 2023, pp. 182–197).

**Fig. 3:**
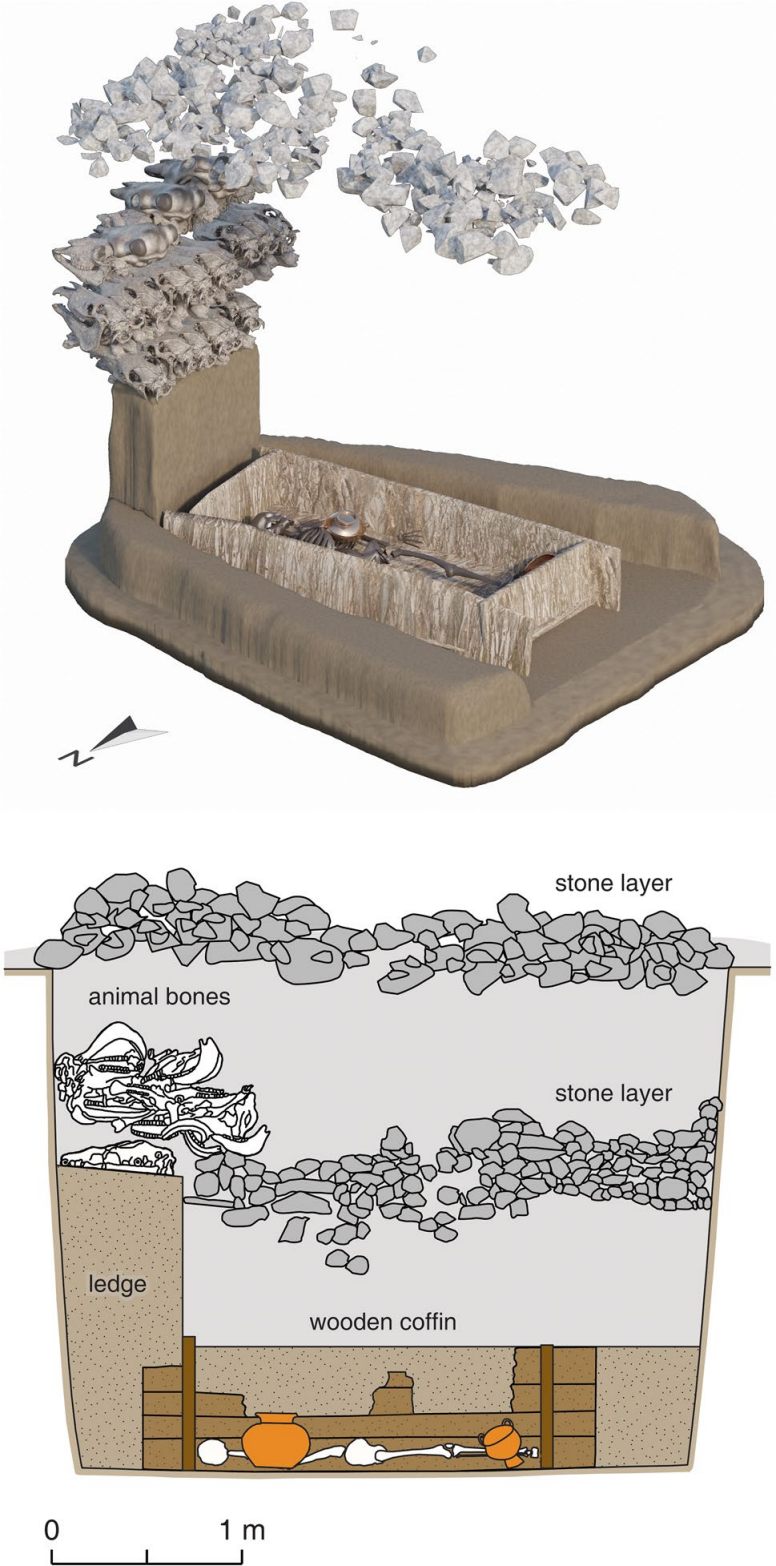
YHM Grave 18. The burial pit shaft was covered by a stone layer. Animal offerings were deposited on an earthen ledge on the eastern side. A second stone layer was at the same height as the ledge. The deceased individual was placed in a wooden frame, probably not jointed, beneath the stone layers. Most grave goods were placed in the frame, around the individual. The 3D reconstruction shows the top stone layer and the inside of the tomb shaft (modified from Beijingshi 2007; 3D model by L. Huan)

**Fig. 4:**
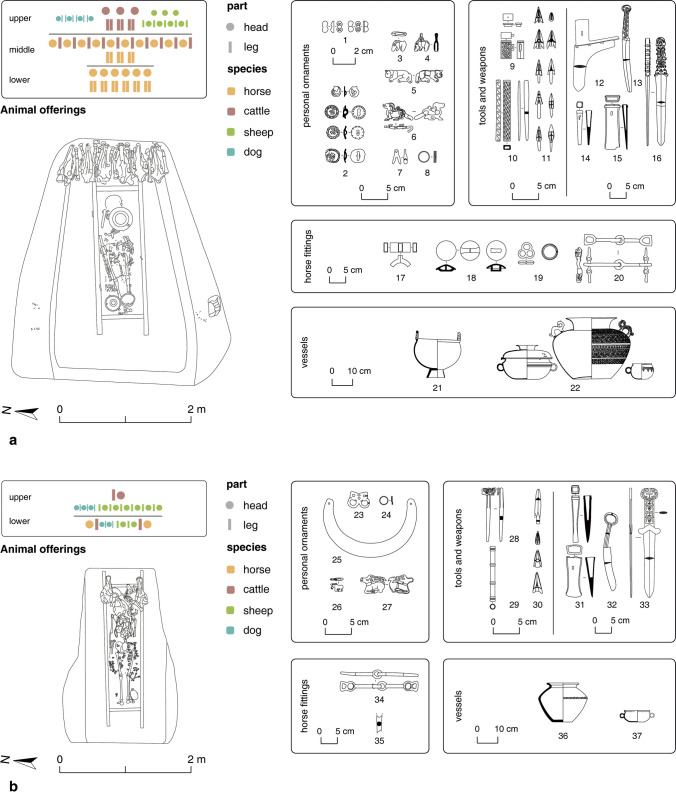
Ground plan of two large graves at YHM: a: Grave 18; b: Grave 174. Both graves are quadrilateral with one enlarged side (trapezoid-shaped). The types and arrangements of the animal remains are demonstrated. Artifacts from the graves are depicted on the right side (modified from Beijingshi 2007; created by L. Huan and K. Hölzl)

The connections between the Yuhuangmiao culture and the steppes are visible in different aspects: grave construction, burial rituals, and material culture. The Yuhuangmiao burials were usually in rectangular shaft (pit) graves. Some also had a single boulder or a layer of limestones above the grave shaft. Inside the graves, animal heads were deposited on a higher level (ledge) near the head of the buried person (Fig. 3). Numerous characteristics at Yuhuangmiao were shared with the slab burial culture, mainly found in today’s central and eastern Mongolia and the Transbaikal region and has been studied most intensively by Tsybiktarov (1998; 2003). However, over the past decades, this cultural phenomenon has not been analyzed in greater detail, and there exists currently no finer internal chronology for the slab burials (ca. 1100–400 BCE). One group of associated burials, the Dvortsov type, was built more elaborately with more animal offerings (Kirillov 1988a; Shao and Yang 2022; Tsybiktarov 1998). Similar burials have been identified in Mongolia recently, where they are called “Multiple Animal Offerings Burials (MAOB),” named after their most characteristic feature. The exact relation between slab burials, Dvortsov-type burials, and MAOB still needs to be determined (Gantulga et al. forthcoming). The MAOB burials and the large Yuhuangmiao burials (e.g. Grave 18) shared numerous traits and were comparable in grave construction (especially shaft graves with a ledge covered by stone layers) and the deposition of numerous animal heads on the ledge. However, unlike in Mongolia and Transbaikal MAOB, where heads of horse, sheep, goat, and cattle were deposited, animal offerings in the Yuhuangmiao graves often include not only these animals but also large numbers of dogs (Online Resource 2). The choice of depositing numerous dog heads points to a local practice (Fig. 4).

Shaft burials are not alien in northeast China as they also occur in burials of the Upper Xiajiadian, the preceding culture, albeit with a center northeast of the Yanhuai Basin, in today’s Liaoning, Hebei, and eastern Inner Mongolia (Linduff et al. 2018, pp. 104–118; Liu 2000; Shelach-Lavi 2005). The Upper Xiajiadian is dated by typochronology to around the final second and early first millennia BCE. Objects and burial patterns of the Upper Xiajiadian culture point to a connection to the slab burials. However, the relative chronologies of the two cultural groups have not been refined and thereby cannot be thoroughly evaluated. Upper Xiajiadian burials were often built with boxes of stones. Some Upper Xiajiadian burials also had wooden frames which were similar to those in the Yuhuangmiao burials (e.g. Xiaoheishigou: Neimenggu et al. 2009, p. 295). Nevertheless, the Upper Xiajiadian burials usually had more stone structures (such as whole stone frames of coffins), while animal offerings were missing in Upper Xiajiadian burials. In the Yanhuai Basin, only a few sporadic finds of Upper-Xiajiadian-type objects have been published (Hong and Wang 2014). The dating of these objects, as well as the relationship between the Upper Xiajiadian and Yuhuangmiao groups in this particular region, remain vague (sites with Upper-Xiajiadian-type objects, see Online Resource 1).

Some objects attributed to the Yuhuangmiao culture are also known from the slab burials, including several types of small ornaments (Fig. 1: objects 8–15; Tsybiktarov 1998). In the slab burial culture, there were also hollow, tube-like objects with a round cross-section, usually regarded as whip handles (Fig. 1: object 16; Tsybiktarov 1998, p. 257, Fig. 70,1.2). In the Yuhuangmiao burials, there were tube-like objects with either a round or a rectangular cross-section (Fig. 4: objects 10, 29). Most of these objects were made of bronze, while a few were made of animal bone. Since the excavators found needles or awls (and occasionally cloth with pinholes) in several rectangular tubes, the rectangular objects are regarded as needle cases. To be cautious, we separate tubes into two groups based on their shape. For those with a rectangular shape, we consider them needle cases following the excavators’ suggestion. For those with a round shape, we consider them a different type of object, possibly a whip handle (under the name “tube”).

Besides these objects, there are also objects not known from slab burials but have their closest analogies in the Arzhan-2 burial in Tuva (Chugunov et al. 2006, 2017). These are small decorations (sliders, Fig. 1: object 5; Chugunov et al. 2010, Pl. 29),^2^ golden pectorals (Fig. 1: object 4; Chugunov et al. 2010, Pls. 65, 87), bronze cauldrons (Fig. 1: object 7; Chugunov et al. 2010, Pls. 84, 85), and small boar figurines (Fig. 1: object 3; Chugunov et al. 2010, Pl. 16; similar but bear figurines in Kazakhstan: Roberts 2021, pp. 30, 37). The range of objects, their number and the closeness of the morphology point to possible immigrants (Rawson 2023, p. 197; Shulga 2015, pp. 116–124, 294–296). In addition, some Yuhuangmiao hooks or buckles (Type A, see examples in Fig. 9) are similar to those from present-day Kazakhstan and the Altai (in this paper, we put them under the large type group of hooks) (Kazakhstan: Bernshtam 1952, Pl. 128; Margulan et al. 1966, Pl. 8; Altai: Rudenko 1960, Pl. 19; see also discussion in Höllmann et al. 1992, pp. 102–104). Although those artifacts in Yuhuangmiao clearly show connections to the Sayan region and present-day Kazakhstan, the burial construction differed greatly. Kurgans (burial mounds) were typical in Sayan and Kazakhstan, while in Yuhuangmiao there were shaft graves.

Central Inner Mongolia is also a region of interest in cultural interaction. Several sites here, including Maoqinggou (MQG), also had shaft burials with multiple animal deposits (Höllmann et al. 1992; Neimenggu 1986; Shulga 2015, p. 297). Objects similar to those from the slab burials (Fig. 1: objects 18–20, 23, 24), Arzhan-2 (Fig. 1: objects 21, 22), and Kazakhstan (Fig. 1: objects 26) also appeared here. Possibly, cultural contacts between the Yanhuai Basin and the steppes may have gone through central Inner Mongolia, for which a higher resolution in dating would be desirable. Based on current typochronological studies, the oldest burials of MQG date to the second half of the seventh century, the youngest to the late fourth century BCE (Höllmann et al. 1992, p. 29; Pan and Tan 2018; Yang 2004, p. 85). While relations to the Siberian Tagar culture are visible, no direct relations to Arzhan are reflected in the MQG artifacts. In other words, the steppe-related elements in the early stage of the Yuhuangmiao culture (e.g. 600–550 BCE, see later detail of the absolute dates) may have come from the steppes directly. In later periods, however, there may have been more emphasis on direct interactions between central Inner Mongolia and the Yanhuai Basin.

**Fig. 5:**
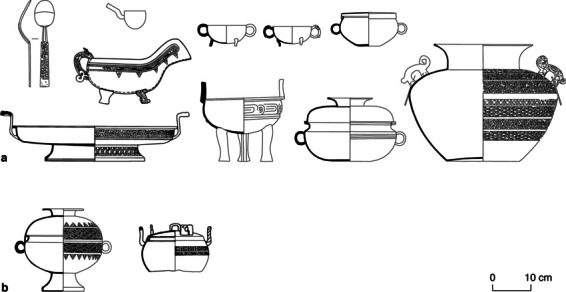
Ritual bronze vessels from two graves: a: YHM Grave 2; b: XLG Grave 1. These vessels are used by researchers to determine the absolute dates of the graves (modified from Beijingshi 2007, 2010; created by L. Huan and K. Hölzl)

The relations between Yuhuangmiao and the Chinese Central Plains are only visible through objects and probably very limited features in the burial ritual. One such instance is in YHM Grave 2 where a group of Central-Plains-style bronze vessels were found near the head of the deceased. They may have originally been placed in a wooden box which was not well preserved (Beijingshi 2007, p. 284). This practice was common for burials following the Central Plains tradition (e.g. Liulihe: Beijingshi 1995). Some Chinese ritual vessels, weapons, and jade objects also appeared in the Yuhuangmiao graves (Fig. 4: objects 22, 37; Fig. 5; Beijingshi 2010, p. 524). In contrast to the steppe-related objects used regularly in many graves, objects with an origin from the Central Plains were only found in a few graves, most of which were much larger than others, and each grave usually had only one bronze vessel. That they contain only a single vessel is a stark deviation from the practices on the Central Plains (Teng and Zhang 2014; Zhu 2009, p. 2120). Only in a few graves, multiple ritual vessels were found (YHM Graves 2, 18, and 250; XLG Grave 1), which is comparable to those from the Central Plains (in comparison with vessels from Luoyang Zhongzhoulu, Fig. 1: object group 27). As Rawson (2023, pp. 186, 191) has pointed out, most people buried with Chinese vessels did not take over the ideology, rituals, and practices associated with these ritual vessels in the Central Plains, while the objects remained exotic.

**Fig. 6:**
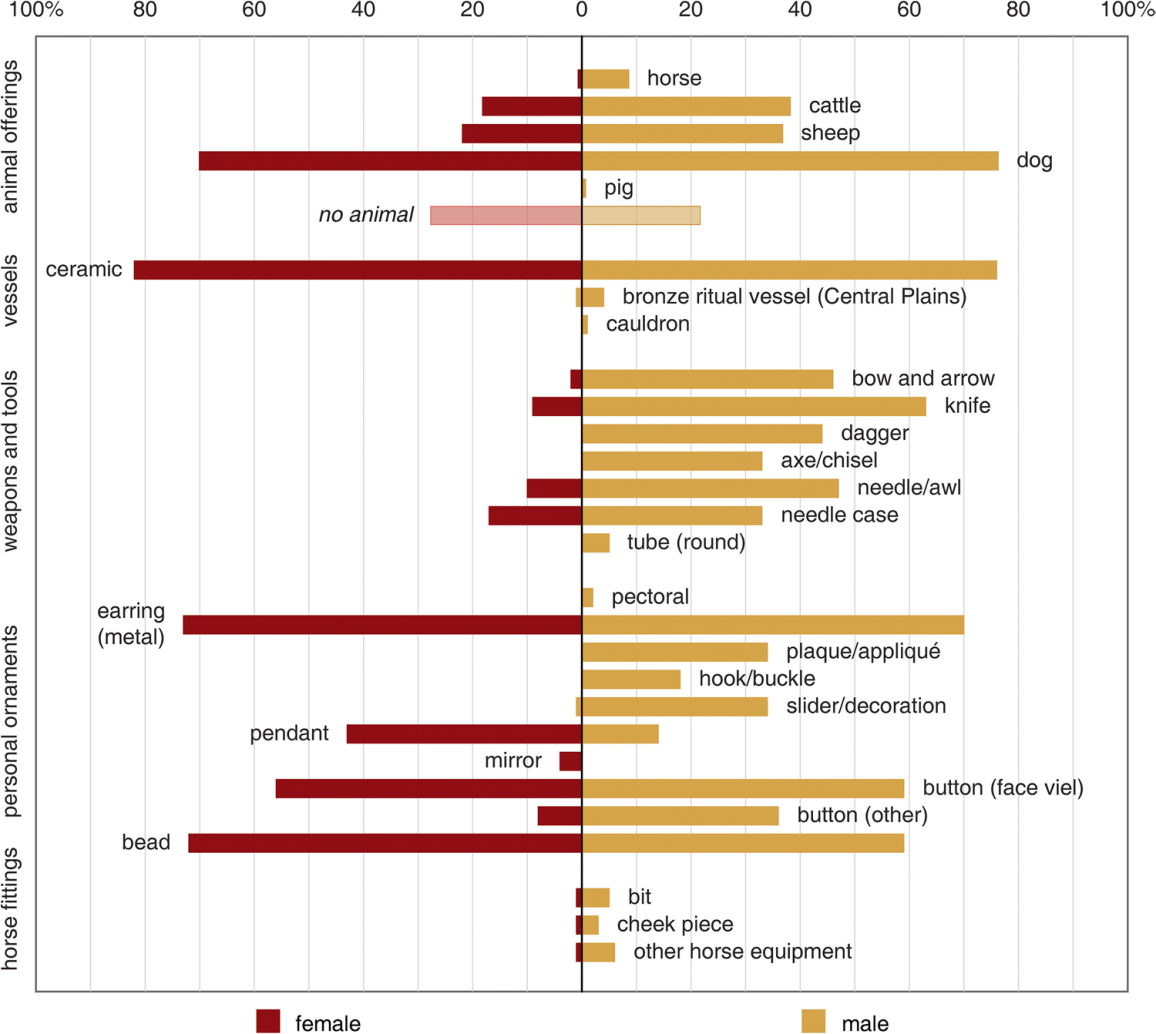
Occurrences of major types of objects and animal offerings in the YHM graves by the identified biological sex of the deceased individuals. Since the animal offerings in many graves have been disturbed, only intact graves are used for the comparison. The occurrences are shown as the percentages of the total intact grave numbers (sample size: 137 female graves, 152 male graves; data: Beijingshi 2007; Online Resource 2; created by L. Huan and K. Hölzl)

In addition to the external cultural connection, burial patterns and objects of the Yuhuangmiao graves also suggest remarkable intra-community diversity and generational changes (Online Resource 2). Three significant differences were: 1) between large and normal-sized graves, 2) between female and male graves,^3^ and 3) between horse-related and non-horse-related graves. At YHM, a few large graves had many more objects and animal offerings than the others (Jin 2004). Regarding the gender differences, some types of objects almost only appeared in the graves of one particular gender (Wang 2022). Objects such as daggers, arrowheads, knives, awls or needles and their cases, bronze ornamental plaques, and hooks were primarily found in male graves. Female graves usually had fewer objects and also fewer gender-specific objects (objects associated with females in Yuhuangmiao include large earrings, bronze mirrors or bosses, and a few types of small beads) (Fig. 6). Nevertheless, the sizes^4^ of the graves do not differ considerably between male and female individuals (median: 2.60 m^3^ of the 150 intact male graves vs. 2.37 m^3^ of the 137 intact female graves). One particular phenomenon reflecting both gender and status was the presence of horse remains and horse fittings. In East Asia, horses first appeared in burial rites around the last quarter of the second millennium BCE, a clear indication of the cultural contact between the steppes and the Central Plains (Rawson et al. 2021). At YHM, elements of horse-gear (such as cheek pieces and bits) appeared in 12 graves, all with horse remains, of which 11 belonged to male individuals. Graves with horse remains were also relatively larger than others (average: 12.92 m^3^ of those with horse remains vs. 2.28 m^3^ of those without horse remains). These patterns suggest that there may have been a group, mainly males, who controlled more social power and resources than the rest, while horses were considered crucial to the status and identity of this small group.

One major obstacle to investigating both the external cultural contacts and the internal social structure is dating. Due to various limitations, the dates of key sites of the Yuhuangmiao phenomenon are still unclear. This uncertainty concerns not only the absolute dates but also the temporal sequencing, making it difficult to reconstruct changing social dynamics. In this paper, we present the current understanding of dating, followed by a new chronological framework based on the typological study of the objects from the three published cemeteries. With this chronology, we review the issues of first, what exactly was the role and representativeness of the steppe elements in the studied community; second, how did these cultural elements and people’s lifestyles change over time? Furthermore, we tentatively suggest a new model for understanding these agro-pastoral communities in the middle zone of Northeast Asia between the steppes and the Central Plains.

## Current dating

**Fig. 7:**
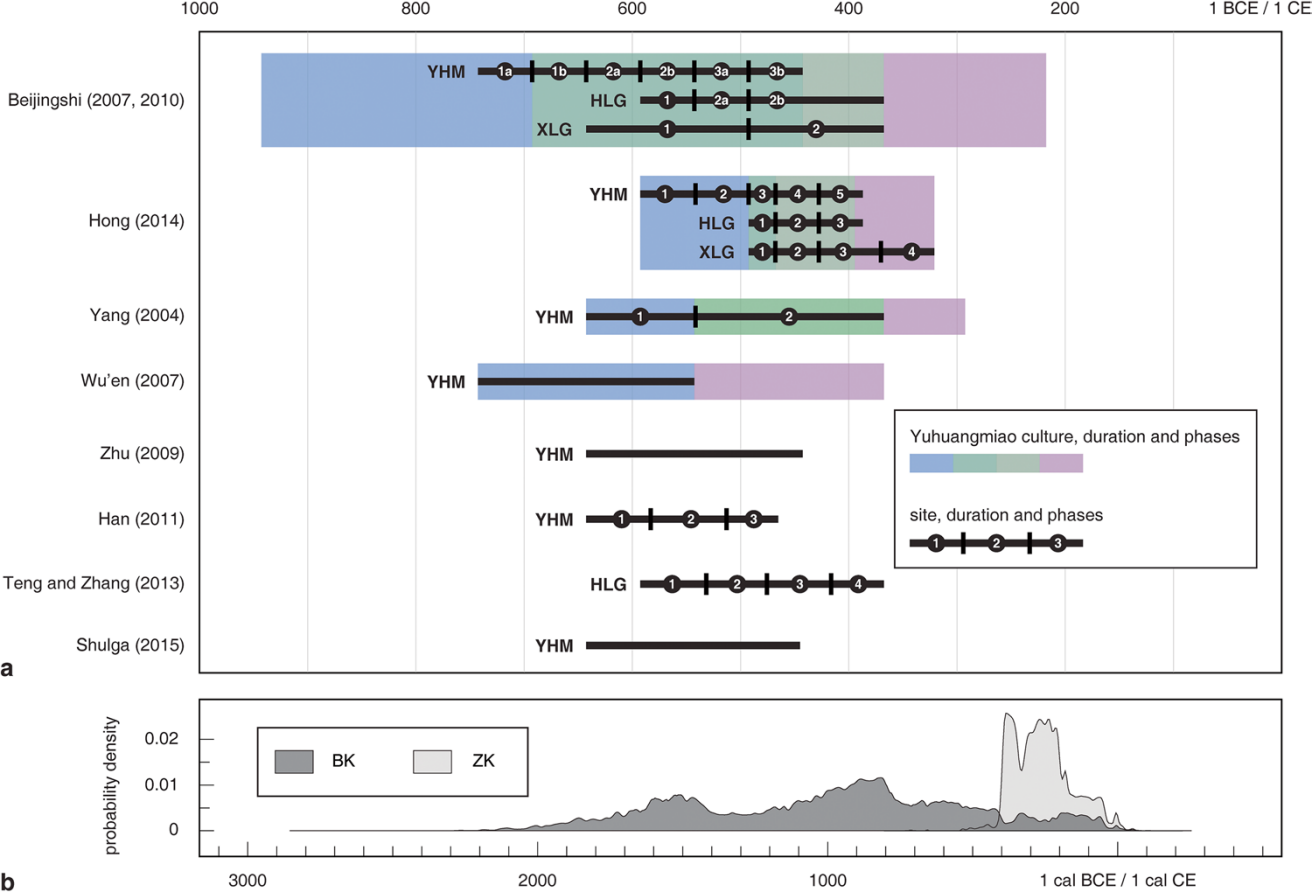
Current relative and absolute chronologies of the Yuhuangmiao culture and sites. a: relative phases and timespans proposed by researchers based on the typochronology. b: accumulative.^14^C dates of YHM remodeled from the original results produced by two labs (BK: Peking University; ZK: IA CASS) (data: Beijingshi 2010, p. 788; Online Resource 2; created by L. Huan and K. Hölzl)

The current relative chronologies of the Yuhuangmiao culture have been established based on two approaches: typochronology and stratigraphy. The typochronological approach uses the morphological changes of objects (such as ceramic vessels and bronze daggers) to determine the internal phases (Kim 2021; Teng and Zhang 2013; Zhu 2009, pp. 2122–2125) (Fig. 7; Online Resource 2). Besides object-based dating, stratigraphic evidence is available at several sites. At YHM, there is a layer of debris which has been dated to 2600–2500 BP based on the geological evidence at other sites near Beijing. Most northeastern graves of the cemetery are covered by this debris layer, while graves in the southeastern part of the cemetery cut through the debris layer. The succession of the layers suggests that graves in the northeastern section are older than those in the southeastern section, providing a coarse division of two phases.

For the absolute dates, most researchers consider YHM, HLG, and XLG to date between 600 and 400 BCE (see citations in Fig. 7a). The start date is determined by two criteria. The first is the presence of Chinese bronze vessels in YHM Graves 2 and 18 (Figs. 4a, 5a). By comparing these objects with similar ones from Luoyang (Fig. 1: object group 27), researchers date these vessels to 600–550 BCE (Hong 2013; Teng 2002; Zhu 2009, p. 2121). The second criterion is the presence of objects similar to those from Arzhan-2, especially Grave 5 (Fig. 1: objects 3, 5). This grave has been precisely dendrodated to 640–600 BCE (Chugunov et al. 2010, p. 172). The end date of the three Yuhuangmiao culture sites can only be estimated based on the ritual vessels from XLG Grave 1 (Fig. 5b). Compared with similar objects from the Central Plains, this vessel has been dated to 450–400 BCE (Hong 2014, p. 69). Regarding the dating of Yuhuangmiao and neighboring cemeteries, the variation among different researchers is mainly caused by a discussion of whether a specific site should be included or excluded from this cultural group. The original excavators of the three sites suggest a long duration of the Yuhuangmiao culture, with the three sites roughly in the middle of the period (Beijingshi 2007, 2010). By contrast, most other researchers argue that sites attributed to the early stage of the Yuhuangmiao culture belong to a different cultural group (Fig. 7, the difference between Beijingshi 2007 and others). Hence, these researchers date the Yuhuangmiao culture to around 750–350 BCE, while the Yuhuangmiao site was in the relatively early to middle stages of the culture.

Nineteen radiocarbon dates have also been generated based on the wood and bone remains from YHM, HLG, and XLG (Fig. 7b). The dates were produced by the laboratories of two Chinese institutes (BK: Peking University, in 1990–91; ZK: IA CASS, in 2000–01; Online Resources 2, 3). Nevertheless, most results do not fall within the range suggested by the typochronological methods. Dates of the samples from the same graves generated by two laboratories also do not agree with each other (dates by BK are uniformly older than those by ZK) (Beijingshi 2010, p. 788). For these reasons, the dates are widely considered questionable. According to our communication with the excavating institutions, there is no plan to redate the materials. Hence, the absolute dates of the Yuhuangmiao culture and the internal phases are established mainly by relative dating methods.

So far, most studies on the Yuhuangmiao culture have only been available in Chinese, with the exception of a study by Shulga (2015) in Russian. Shulga divided the YHM cemetery into sections and suggested that each part of the cemetery was used for a discrete period of time. By referring to the stratigraphy and examining the typological changes of artifacts across the sections, Shulga suggests a very fine temporal sequence for the whole cemetery in eight phases, while each section belongs to one phase (Online Resource 2). One problem with this model is that the existing sequence stratigraphy can only suggest a two-phase chronological sequence. Any finer division of the sequence is only based on the assumption that the cemetery was constructed and used section by section, which still needs to be confirmed. Besides, having as many as eight phases also makes it difficult to compare the typological differences of the objects. We will discuss this issue in the final discussion.

## Materials and methods

Considering the importance of understanding the group of Yuhuangmiao given its close ties with neighboring and distant regions (such as Tuva), we aim to develop a relative chronology that allows us to study the socio-cultural changes within that community. We employ contextual seriation (presence vs. absence) instead of frequency seriation since in most graves only one object from each type was found. We use correspondence analysis (CA) to check and identify potential biases in the dataset. This method was first proposed in the 1930s and introduced into archaeology in the 1970s (Hirschfeld 1935; Hill 1974; Doran and Hodson 1975; Bølviken et al. 1982; Madsen 1989). It is now a common practice in typochronology (Baxter 1994; Shennan 1997; Groenen and Poblome 2003; Lock 2003; Baxter and Cool 2010). CA provides a seriation order by comparing the closeness of the object types (Beh and Lombardo 2014). The method can also suggest whether the closeness is likely to reflect a single factor (such as time) or multiple factors (such as both time and gender) (Hill and Gauch 1980; Jensen and Nielsen 1997). In this study, we use TOSCA, an online seriation tool developed by Hinz (2018), to sort the graves based on the CA results. The whole process contains four steps:

- devising a typology for the objects
- Identifying and separating the graves by gender
- producing the seriation with the object types
- checking with CA for biases in the dataset and controlling whether a temporal sequence underlies the identified groups (parabola-shape, see later explanation)

We used YHM as the benchmark since the site has the most abundant graves and objects. Although we aim at a temporal sequence for the whole cemetery, the seriation and correspondence can only be applied to the male burials. Most objects related to female burials, such as large earrings and bosses/mirrors, are morphologically very homogeneous and thus cannot be further classified. However, this also means that potential gender bias would not be significant in the seriation even if we process all the graves in a batch. We chose ten types of objects of various categories, including weapons and tools (daggers, knives, metal arrowheads, needle cases, tubes), personal ornaments (hooks, plaques, sliders, and other small decorations), and horse fittings (bits) (Online Resource 2). These objects are widely distributed in the graves, while morphological differences are also recognizable. Unlike previous studies, we do not include ceramic vessels since the corpus looks homogeneous, and the line drawings do not allow us to identify differentiations. Bone/antler arrowheads are also not included due to the poor preservation and the ambiguity of the drawings. Furthermore, it needs to be pointed out that classification is a subjective process. Even when statistical methods or clustering are applied, the results are prone to human bias.

Two runs of seriation were processed. The first run includes all graves and objects that are not single-occurrence cases (i.e., a type with only one sample or a grave with no or only one defined object). In the initial result, some object types are not sensitive to changes of graves or vice-versa. These types/graves are excluded since they are not sensitive to temporal changes (Online Resources 2, 4). Two runs of CA were also processed. Normally, an ideal dataset should generate a parabola-shaped distribution of the CA result (also known as the “arch effect”) (Hill and Gauch 1980; Jensen and Nielsen 1997; Kendall 1971). This shape suggests that time is the main factor reflecting the differences, instead of other factors such as age or wealth. Our results suggest that most objects and graves follow the parabola-shaped distribution (Online Resource 5). Therefore, we can confirm that the seriation based on the selected types and graves reflects a temporal order. The two outliers are the Type F dagger (with a disk-shaped end and a fuller) and the Type D hook (with many round holes that may be used for inlaying other materials). As previous studies suggested, outstanding CA results indicate that the objects are particularly associated with a few graves. In other words, the presence/absence of these objects does not reflect a single temporal factor (Doran and Hodson 1975; case study: Stadler 2008). For the seriation, these two types are excluded. After excluding these items and new single-occurrence cases, the second run of seriation and CA are processed as our final results.

Based on the seriation, we divide YHM graves into three groups. The stratigraphic sequence of the site is then used to determine the direction of the seriation (Beijingshi 2007, pp. 12–14). Graves included in the seriation are then mapped on the cemetery plan. In this step, single types are drawn together with the burials in the seriation. Since the graves of the other two cemeteries, HLG and XLG, were relatively object-poor, graves from the two sites are dated relatively to one of the YHM groups/phases by the same object types.

## Results

With the above methods, 75 out of the 400 graves from YHM are included in the final seriation results. Since most types of objects are only associated with one sex, we do not separate the graves by gender. Among the graves, 70 of the individuals were identified by the excavators as males; one was identified as a female. The biological sexes of the other four individuals were not identified (Online Resource 2).

**Fig. 8:**
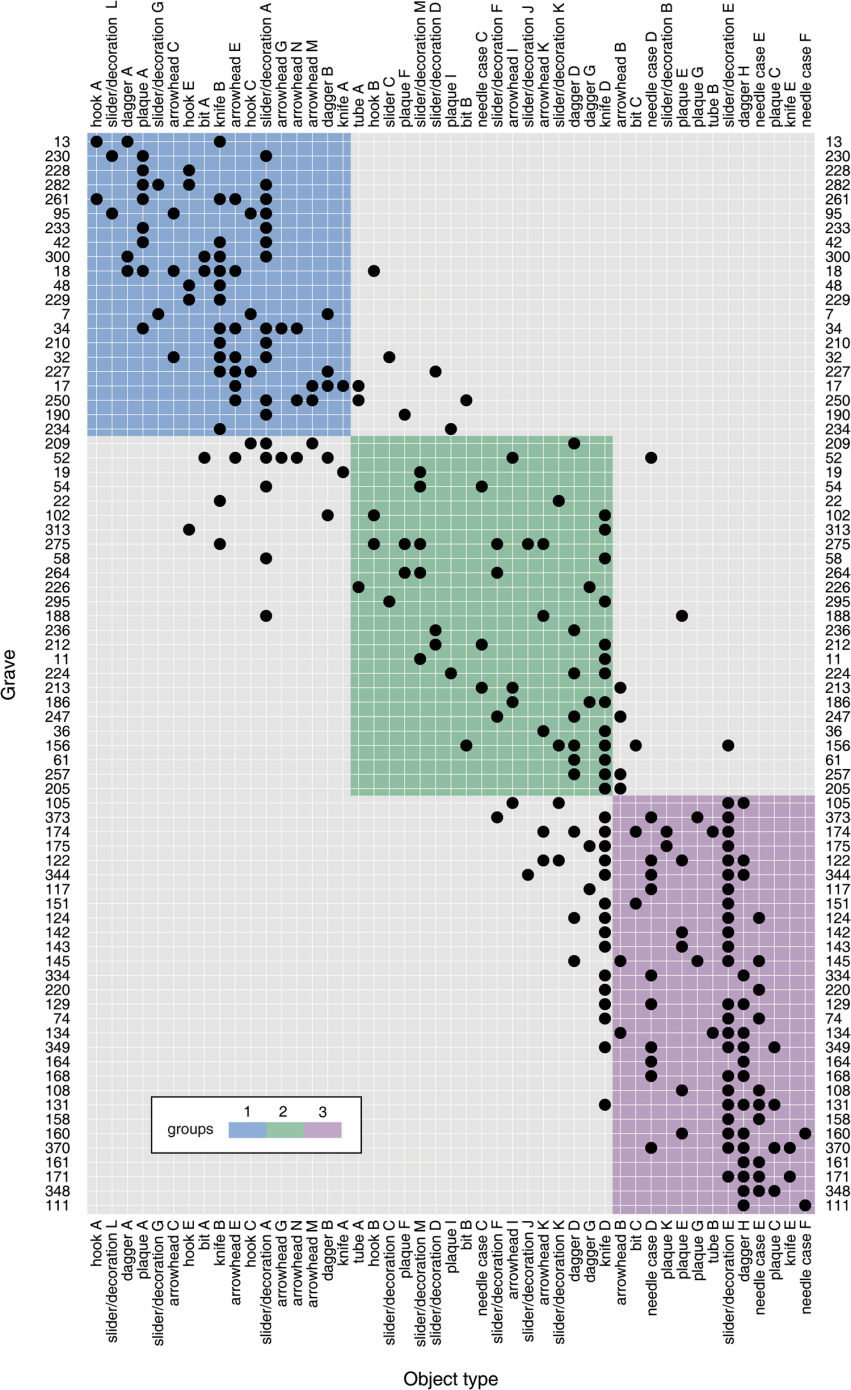
Seriation of graves from the Yuhuangmiao site based on correspondence analysis (program: Hinz 2018; dataset and object typology: Beijingshi 2007; Online Resource 2; created by L. Huan and K. Hölzl)

**Fig. 9:**
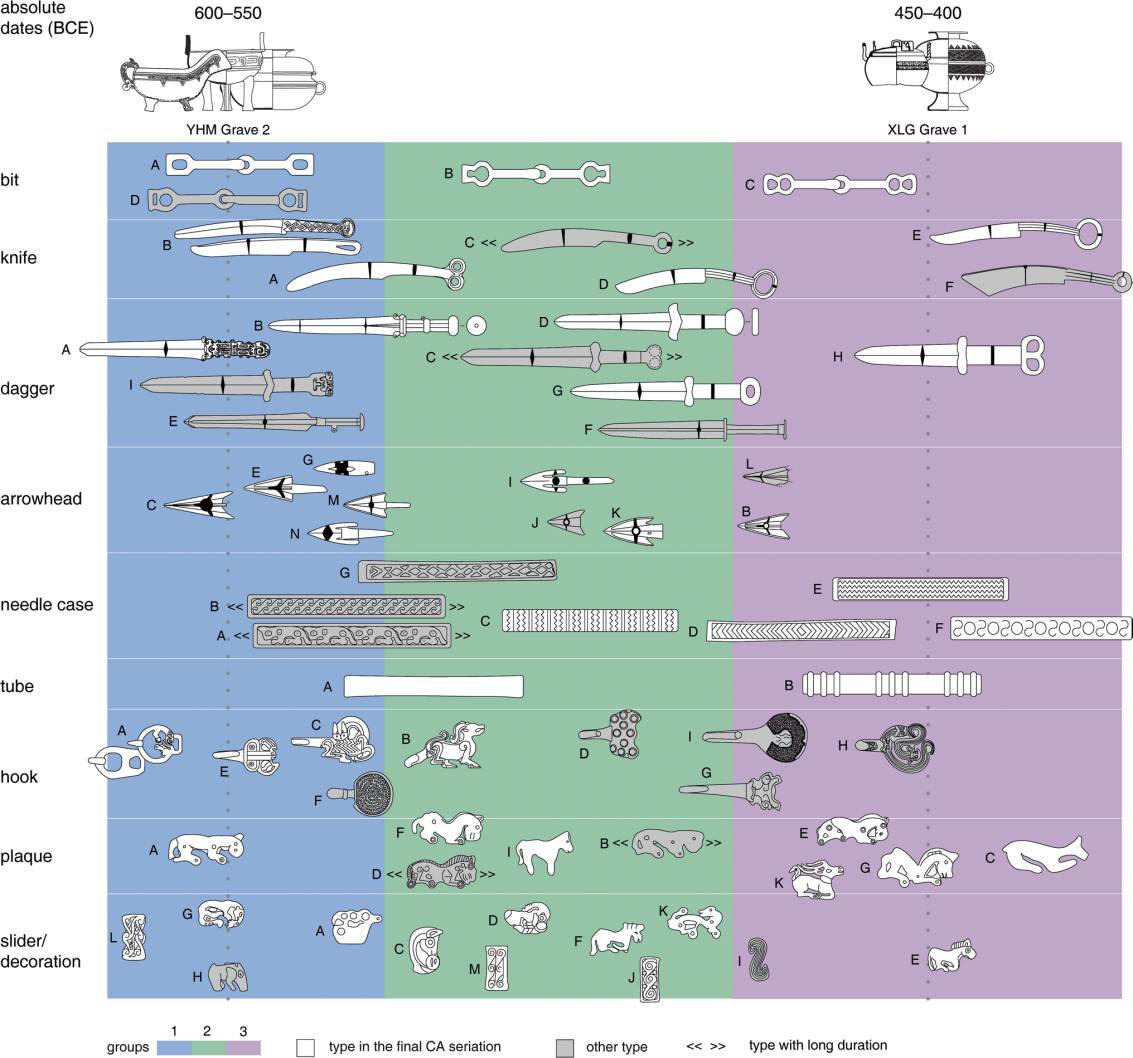
Object types in the Yuhuangmiao seriation. The three groups are determined according to the occurrence of the objects in Fig. 8. Object types with relatively certain dates are added, including those with relatively long durations in the initial seriation result (Online Resource 4). The relative positions of the Chinese-style ritual vessels, which are used to determine the absolute dates (Fig. 5), are also illustrated based on the suggestions by Hong (2014) and Zhu (2009) (created by L. Huan and K. Hölzl)

**Fig. 10:**
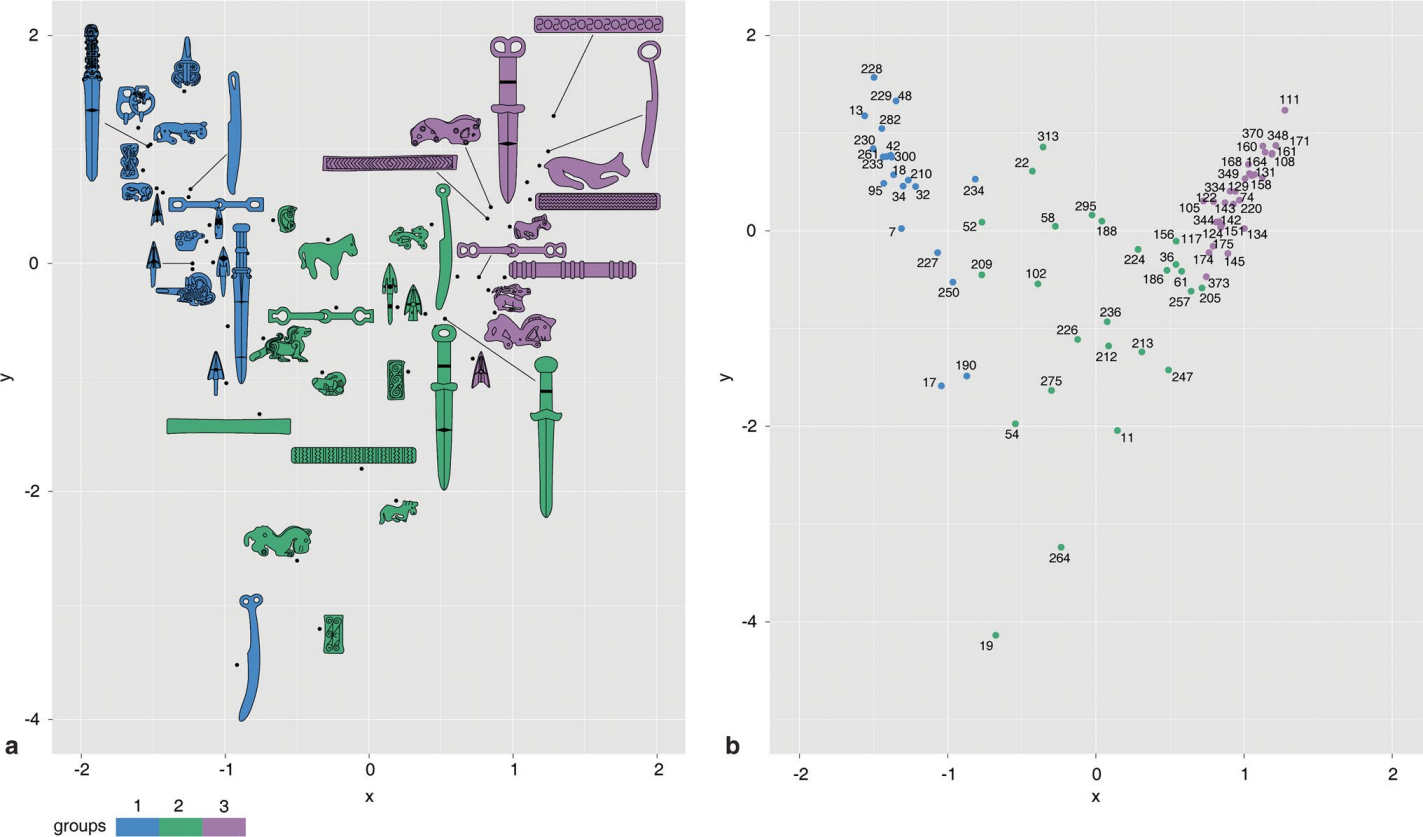
Correspondence analysis of the first and second axes. a: type; b: grave. The Scatterplots with a parabola-shaped distribution suggest that the style changes were due to a single factor such as time (program: Hinz 2018; created by L. Huan and K. Hölzl)

In the final results, we divide the graves into three groups (Fig. 8). The first group is represented by Graves 18, 230, and 250 (illustrations of graves with objects, see Shulga 2015, pp. 189–191, 197–201), with typical objects such as the Type A bits, Type A daggers, Types A, C, and E hooks, Type A plaques, Types G decorations, and Type L sliders. The third group is represented by Graves 151 and 174 (Shulga 2015, pp. 212–213, 220–221), with typical objects such as Type E knives, Type H daggers, Types D, E, and F needle cases, and Types C, E, and G plaques. Between them is the second group, represented by Grave 156 (Shulga 2015, pp. 216–217). As previously mentioned, some object types are left out in the seriation due to their long duration or single-occurrence scenarios. We add some types back to show a complete range of typological changes (Fig. 9; Online Resource 2). The CA results of the graves and object types follow the parabola-shaped distribution, suggesting that the seriation most probably reflects a temporal change (Fig. 10).

**Fig. 11:**
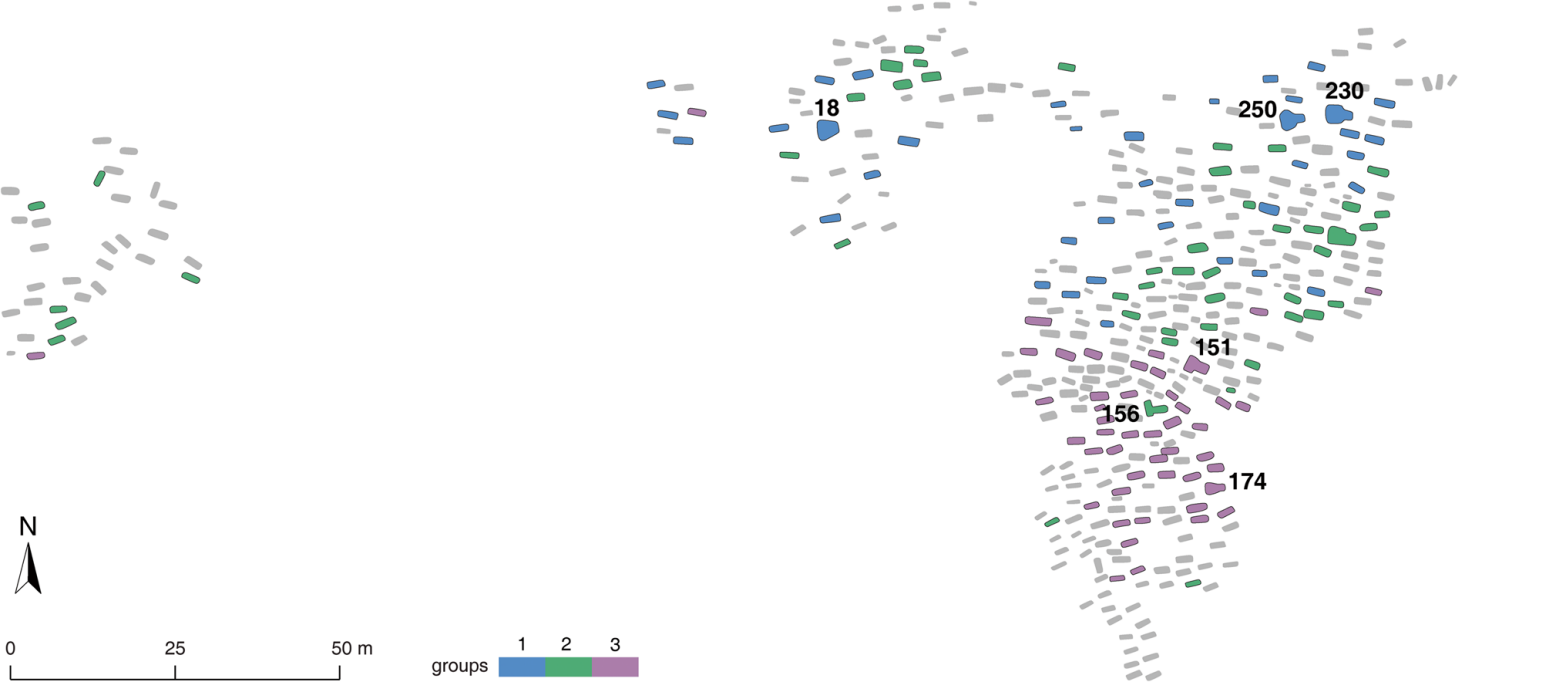
Plan of the Yuhuangmiao cemetery. Graves that are covered by the seriation order are color-coded. Large graves mentioned in the text are marked on the plan (data: Beijingshi 2007; Online Resource 2; created by L. Huan and K. Hölzl)

The graves from the seriation and single occurrence cases are now marked on the ground plan of the cemetery (Fig. 11). As the figure shows, graves in the northeastern and southeastern sections belong to two groups. The result confirms the previously suggested rough expansion of the cemetery in the course of its use. The western graves belong to a relatively separated cluster. These graves are linked to Groups 2 and 3. With the stratigraphic information from the site, we can determine that Group 1 represents the oldest phase (Phase 1), while Group 3 represents the youngest phase (Phase 3). In addition to the general expansion, as previous studies have suggested, our model allows for a more refined interpretation that the graves were constructed in several clusters. For example, in Phase 1, Grave 18 was built as a central grave in the north-central part of the cemetery, while Graves 230 and 250 were two in the northeastern part of the cemetery (Fig. 11). All three graves belonged to male individuals. They were surrounded by several relatively small graves. For the younger phases, one cluster was centered around two male burials, 151 and 156, while an almost contemporaneous cluster, also centered around a male burial, 174, was built in the southwestern section. The cemetery arrangement based on these large male burials indicates that these individuals may have had central roles in the community.

For HLG and XLG, many objects are of the same types as those from YHM. By choosing some of these object types, we assign most datable graves from HLG and XLG to Phases 2–3 of YHM (Online Resources 2, 6). The internal chronology agrees with the stratigraphic sequences at both sites (Beijingshi 2010). The seriation results also confirm the previous suggestions of the absolute period of the three sites. The first phase began around 600 BCE, as vessels from YHM Graves 2 and 18 (both in Group 1) date to 600–550 BCE. The third phase ended around 400 BCE, as vessels from XLG Grave 1 (first half of Group 3) date to 450–400 BCE (Fig. 9). Therefore, the whole period of use for the cemetery can roughly be dated to around 600–400 BCE.

## Discussion

The proposed temporal sequence confirms some previous suggestions (Hong 2014; Zhu 2009), but our results also reveal further characteristics of the community. In this section, we will first compare our chronology with the existing ones. Then, we will discuss the potential implications revealed by the new chronology.

### Comparison with previous relative and absolute dates

Our CA-based seriation and previous non-CA methods provide relatively close phases of typological changes that support each other but with two main differences. First, our model divides the whole period into three phases, while previous models often suggest four or five phases and even sub-divisions in each phase, which, in our opinion, is too fine (Fig. 7). Second, we chose fewer object types than most previous models did. The reason for choosing fewer phases and object types is the sample size our statistical method requires (there should be sufficient numbers of objects in each type and phase). Considering the relatively short duration of the three sites (about 200 years) and the relatively subtle changes in object types, we would argue that having three instead of four or five phases (or even ten) is more suitable.

With the seriation order, we can also reconsider the existing ^14^C dates from the three sites. We agree with most researchers that the long duration suggested by the ^14^C dates (Online Resource 3) should be rejected. A further comparison of the graves with the ^14^C dates and those covered by the seriation order shows that, although the dates produced by the two laboratories depart from each other, the relative successions of the graves are similar and are also close to what our seriation model suggests. The reason for the inaccurate ^14^C dates is impossible to confirm. However, as a general view of the radiocarbon dating method, the issue of the Hallstatt plateau is worth noting. This refers to the effect that the flat plateau in the calibration curve draws all calibrated dates to the same range (Plicht 2004). The Hallstatt plateau affects samples whose actual dates fall between ca. 800 and 400 BCE. In future studies, other methods, such as dendrochronology and high-resolution relative dating (e.g. based on Chinese ritual vessels with well-dated inscriptions), need to be considered when dealing with materials from Yuhuangmiao or contemporaneous sites.

### Interpreting changes in burial patterns

**Fig. 12:**
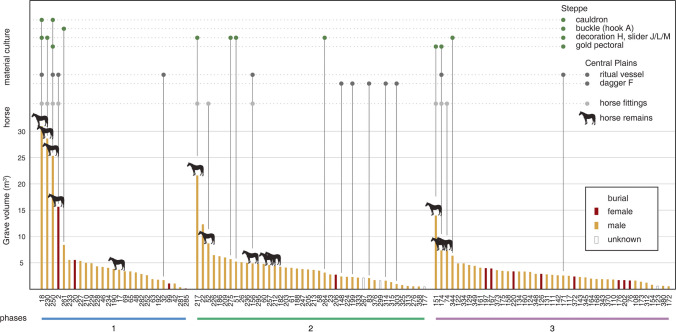
YHM intact graves ordered by group (phase) and grave size. Most horse offerings and horse fittings were associated with a few large graves and often adult male individuals in each group. Steppe-related object types first emerged in Phase 1. Over time, the size differences between large and normal graves decreased (data: Beijingshi 2007; Online Resource 2; created by L. Huan and K. Hölzl)

Based on our chronology, graves belonging to each phase can be further divided into two groups: the relatively large graves and the remaining normal-sized ones (Fig. 12). As the size reflects the efforts needed to construct a grave, it is often used as a proxy for the social status or rank of the individuals (Brown 1971; Flad 2001; O’Shea 1984; Trigger 1990). At YHM, large graves were usually over 5 m^3^. Individuals buried in large graves were predominantly males. Most horse remains, as well as horse fittings and elaborate adornments, also appeared only in the large graves. As previously discussed, the construction of these graves (shaft grave, ledge, covered by stone layers) and the burial rite (with numerous animal offerings on a ledge above the deceased) point to the central Mongolian Steppe (MAOB burials), while objects in a few Phase 1 large graves (such as Fig. 1: object 5 and Fig. 9: slider L) suggest a link to further northwest, to Arzhan-2 in Tuva. Therefore, the Yuhuangmiao community may have emerged due to the direct cultural contact with the steppes. A likely scenario is that individuals with strong ties to Arzhan-2 relocated from the steppes (i.e. Mongolia). In Phase 1, we can also observe Central Plains bronze vessels in large graves. These objects indicate that, from the beginning, the elite group also had connections to the Central Plains.^5^ In addition, although some Phase 1 objects are analogous to those from the steppes, some detailed designs are quite different. For example, the handles of the cauldron from YHM Grave 18 are twisted, while the foot of the cauldron from Grave 250 reminds of a stand of Chinese vessels (both cauldrons, see Beijingshi 2007, p. 912). These details are untypical for steppe cauldrons (e.g. Arzhan-2 cauldrons: Chugunov et al. 2010, Pls. 40, 70; see also Guo 1999). The handle decoration of the dagger from Grave 18 (Fig. 4: object 16) also points to a local design. These hybrid objects point to steppe links but a local fabrication.

In Phase 2, large graves continued but were in general smaller than those in the earlier phase. Horse offerings and horse fittings appeared in large but also some medium-sized graves. In Phase 3, although the horse-related graves were still larger than others, the size difference between large and normal-sized graves was not as remarkable as in Phase 1. Meanwhile, some steppe-related objects that were probably used to confirm their privileged status (such as gold pectorals) continued to be used. These burial patterns, together with the previously discussed cemetery layout, suggest that social power was exercised by and transferred among some male individuals who continued to pay tribute to their steppe connections. However, the importance of displaying power or status/rank in the burial rite through conspicuous consumption, as well as objects with steppe connection, seems to have faded over time. This trend can also be observed in the changes in object design, as most objects belonging to Phase 3 had more simplified designs than those from the first two phases. As an example, the abundant motifs of animals in Phase 1—typical for steppe fashion in eastern Eurasia (with the exception of the slab burials)—were mostly replaced in Phase 3 by geometric motifs (Fig. 9). This can be observed with the needle cases, but also with the daggers.

For objects that originated in the Central Plains, Phases 2 and 3 show differences in the display in the funerary arena: In Phase 1, we find several large ritual vessels, especially in large burials (especially Graves 2 and 18). In some large Phases 2 and 3 burials, however, only small ritual vessels and only a single piece were placed. This may suggest that either the necessity of such a large display of ritual vessels lost importance over time or the access to such vessels no longer reflected the high social power or status of the individuals. Meanwhile, some other objects indicate a continuity of connection with the Central Plains. For example, the Type F dagger appeared in several Phase 2 graves. Although daggers in general have emerged in the steppe zone, daggers of this particular type, with a flat, round-shaped end and a fuller (a raised ridge), first appeared in the Central Plains around 700 BCE (Zhu 2009, p. 423). Scholars have suggested that this type of dagger was probably introduced into the Yan Mountains around the Yuhuangmiao time (Li 1982; Lin 1962; Shao 2005; the outstanding CA result also points to this possibility: Online Resource 5).

In previous studies, researchers often consider the Yuhuangmiao culture a discrete group of nomad people (Shulga and Shulga 2020). This is mainly due to the historical texts, especially the Zuo Tradition (*Zuo Zhuan*, first complied around 350 BCE), which report that Yan, one of the Chinese states around today’s Beijing, was constantly attacked by people, known as the Rong, coming from the other side of the Yan mountains. Rong (meaning “the warlike ones”) is an exonym used by the Chinese writers and likely refers to several ethnic or cultural groups (Lin 2002, 2017; Yang 2003). According to the texts, the raids reached a peak around 700–650 BCE. Yan was defeated and sought help from Qi, a state further south, who finally drove the nomadic group away (Rawson 2023; translation of the Zuo Tradition: Durrant et al. 2016). Since the dates of the Yuhuangmiao culture seem to match the recorded conflicts, while the material culture also portrays links to the steppes, many researchers, including the original excavators, consider the Yuhuangmiao culture synonymous with the Rong people. The changing burial patterns, such as the decrease in grave size and horse-related rituals, were then attributed to the decline of the group as it was defeated by the southern Chinese states. While the equation of an archaeological culture with a group mentioned in the written sources comes with numerous problems, the essentialistic view of a discrete archaeological culture also exacerbates this problem. In any case, we caution against a straightforward interpretation of a decline just because the display of artifacts reduced.

We suggest reconsidering the interpretations of both the decline of the Yuhuangmiao community and the Yuhuangmiao culture’s equivalence to a discrete group of people. First, the current archaeological evidence does not support any decline of the local community, such as the abandonment of settlements, the decrease of the local population, or the replacement of the local population by new people. The only plausible evidence is the distribution of a particular pottery type at many sites around the Yanhuai Basin (Guojia 2013) (Online Resources 1, 7). These ceramics are usually associated with the Yan culture of the “Warring States period (ca. 450–221 BCE)” (Hebeisheng 1996). Some researchers suggest that the appearance of these Yan ceramics in the later half of the first millennium BCE reflects that the Yuhuangmiao culture was replaced by the Yan culture (Jin and Wang 2001). However, separating the Yuhuangmiao and the Yan cultures by pottery typology (especially by shards rather than complete vessels) seems problematic. The current data can only show that the Yanhuai Basin continued to be populated during and after the Yuhuangmiao culture period. Any potential demographic change (e.g. the Yan people replacing the Yuhuangmiao people) cannot be inferred from this data.

Second, the “nomadism” of the Yuhuangmiao culture is also an exaggeration rather than a reflection of the local communities’ lifestyle and subsistence economy (this has first been pointed out by Chinese scholars, see e.g. Lin 2002; Yang 2009). The distorted image is partly due to the misleading distinction between agriculturalists and pastoralists, often encouraged by the stigmatization of pastoralists by agrarian states (Scoones 2021; Scott 2017; Di Cosmo 2002; Ventresca Miller et al. 2021), and partly due to the overfocus on cemeteries and grave objects while neglecting settlement data. Although most recognized sites attributed to Yuhuangmiao are cemeteries, a few permanent residential sites with the same material culture have also been discovered. These sites include Daiwangcheng, Baimiao and Xiaobaiyang. The former is a large walled site which may have been built around 700–400 BCE (Du 2019; Han and Jia 1997), while the latter two are open settlements with meter-thick cultural layers and cemeteries yielding Yuhuangmiao-type objects (Zhang et al. 1985; Beijingshi 2015). Due to the traditional focus on objects and cemeteries rather than settlement sites in the local archaeological work, these possible settlement sites have not been comprehensively investigated (for other settlement sites, see Online Resource 7). A recently excavated and published site is Hujiaying, with more than 20 semisubterranean houses, 17 pits, and 11 kilns (Beijingshi 2015). The typochronology suggests that settlement activities lasted from 450 to 250 BCE and may have overlapped with Yuhuangmiao (Beijingshi 2015, pp. 272), since ceramic vessels from the site suggest a clear connection with late-period burials from the Yuhuangmiao culture sites, such as YHM and HLG (Beijingshi 2015, pp. 273–275). These discovered settlements point to a sedentary lifestyle of the local communities, at least for some parts of the people. In addition, although the Yuhuangmiao burial rituals do not expressively show an agricultural component (e.g. agricultural tools were not placed in burials), osteological and stable isotope studies suggest that crops certainly contributed to people’s diet (Chen et al. 2022; Deng 2018; Wei 2004). Due to the limited amount of data, especially the lack of the δ^13^C‰ and δ^15^N‰ data related to the large tombs and horse-related graves, the intra-community diversity or changes through time cannot be observed. Therefore, we cannot know whether the horse-related individuals had a higher consumption of animal protein than others (Online Resource 8). Overall, the data suggests that the mobility of people and their level of meat consumption may have been slightly higher than people from the Central Plains but also significantly lower than pastoralists, such as those from the Mongolian Steppe (Online Resources 9, 10).

For our interpretation, we suggest focusing not on the Yuhuangmiao culture as a whole but on a certain community that can be archaeologically identified (such as the three adjacent cemeteries, since people buried here were likely to reflect a group with a certain level of organization). In this community, people may have always been strategically creating, adopting, and rejecting elements of life from both internal and external sources. These choices, often due to socio-cultural reasons (such as the need to display social power), led to various emphases on rites and objects. In the YHM cemetery, we argue that the observed changes in burial patterns reflect not the decline or retreat of the community and its culture but the decrease in the extravagant expression of steppe-related elements. In its early stage, the newly established community, probably by integrating people from different geographical and cultural backgrounds, may have stimulated its rulers and successors to ensure their power by grand displays on the occasion of the death of a community member. In the late stage, less energy was invested in such activities, possibly as the community became more stable or was sufficiently integrated. The phenomenon of erecting large burials in certain stages of a community’s history has been noticed and discussed by many researchers (Childe 1945; Baines 1989; Trigger 1990; debate and discussion: Brysbaert 2018; Marcus 2003; Osborne 2014). In particular, Kossack proposed a concept called ostentatious burials. According to Kossack (1974), these burials often display factors that are above the local customs, such as exceptional grave architecture, special decorations, goods of high craftsmanship or from foreign lands, display of means of transport and precious costumes (Kossack 1998; see also Brosseder 2009). Those large YHM burials certainly meet these criteria. Kossack argued that, in most cases, the construction of ostentatious burials was not driven by political and social conditions or individual capacities but by the stress of elite members to present their power in a confrontational situation, especially, but not exclusively, when a ranked society encountered other groups (Kossack 1998, p. 33). In the case of the Yuhuangmiao community, those buried in large tombs with horses may also have experienced a similar situation, as their authorities were shaken when the community formed in the Yanhuai Basin. The extravagant burial display, especially in the early stage, was probably a way for the male rulers or heirs to re-negotiate their standing in the community or to construct differences towards their neighbors to the south (since people used mostly steppe-related elements to display the ostentatiousness).

Overall, this study shows that people in these investigated communities were not a group with a homogenous and stable combination of cultural elements, as the concept of archaeological culture often indicates. Among these interregional connections, those to the Mongolian Steppe were more fundamental than to any other region as they went beyond artifacts from there. The grave design (shaft grave with a ledge and stone layer covering) and the burial rite (deposits of numerous animal heads on the ledge above the deceased) indicate the region from where a few immigrants might have come and founded the community. They had strong ties to Arzhan-2 as their accoutrements show. On the other hand, although the artifacts display a wide connectivity, most of them are neither from the Central Plains nor from the steppes directly, but local. Recent fieldwork and archaeometallurgical studies suggest that many objects in the region were produced locally to imitate objects originally from the steppes (He et al. 2002; Wang et al. 2018). Rawson (2023, pp. 193–194) has suggested that some metal resources in this region came from recycling objects produced in the south, i.e. the Central Plains, which future metallurgical studies will possibly elucidate. In the later phases, especially, people tended to display fewer elements from the steppes as well as from the Central Plains. Overall continuity and local elements dominated.

Furthermore, although the historical texts written by Chinese historians often portray the southern Chinese and the northerners as two groups with fundamentally different lifestyles and a hostile relationship, we suggest that the Yuhuangmiao culture was mainly a local phenomenon, typical for the intermediate zone with impulses from north and south, and possibly also neighboring groups in this zone. The nature of the relation may not be visible through the current material evidence. The Yuhuangmiao community may have received what they needed from the Central Plains, such as metal products and/or metallurgical knowledge, while the southern agricultural communities also profited from the knowledge and resources from the north, such as horses, furs, and other pastoral or hunting products (Di Cosmo 1999). Nevertheless, there was one clear difference in the nature of the relations between Yuhuangmiao and the north, the steppes, and between Yuhuangmiao and the south, the Central Plains: while we also see an influx in the burial construction (stone layers, ledges, etc.) and the burial ritual (deposition of animal heads) that relate to the north, from the south, we see an influx of few ritual vessels and objects alone, especially in the early phases. Only in a single instance, in Yuhuangmiao Grave 2, we can perhaps think of a combination of ten ritual vessels (including one drinking vessel, the *lei*, with liquor sediment in it, suggesting the vessel was indeed used to hold liquor). This is the only case in which one may discuss whether an idea was also taken over from the south together with the individual (Zhu p. 2137). For any further evaluation of the relations of local dynamics and external impulses, we need a finer chronology of the slab burials in Mongolia and Transbaikalia as well as a clarification of the relations of MAOB and the slab burial culture and other groups in northern China.

### Conclusions

In summary, this case study offers a new typochronological framework for investigating the cultural and social issues in the Yuhuangmiao community, providing insight into the internal socio-cultural dynamics over time: in the beginning, people from the steppe were the part forming these burials, as the earliest large burials display strong ties to the steppe world, especially Arzhan and the MAOB burials from Mongolia. These ties are visible not only in the artifacts but also in the burial rite (such as numerous animal heads deposited in the burials) and grave construction. The steppe relations, visible in clothing accessories, horse-gear, and weaponry, as well in the ornamentation of local objects, such as the animal motifs (feline, horse, deer), did not only concern the early burials, but continued to play a role throughout the use of the cemetery. However, in the younger Phase 3, these steppe relations faded. A case in point is, for example, the fading animal decoration on the needle cases. On the other hand, objects from the Central Plains in the beginning were a few fancy ritual vessels, which were not used in the Central Plains manner. These rather loose connections to the south continued as well but also faded. However, more simple objects, such as knife F (see above), came to Yuhuangmiao from the Central Plains. The social dynamics highlight that, in the early phase, there were strong needs to construct large burials and display elaborate burial rites, a large number of offerings, and a rich array of objects. These needs, however, seem to have faded over time. Overall, later burials were all smaller and less furnished or even without artifacts than earlier burials. Such a display of elaborate burials and steppe elements may have had its root cause in community building in Yuhuangmiao, such as by helping integrate the community, or in a historical situation of stress (e.g. Kossack). As time passed, the benefits of displaying strong steppe ties in the mortuary arena decreased, the burials looked more equal and differences became less stark. Possibly, the necessity to display social differences became less important and/or may have led to transforming the material culture and the social structure.

Regarding the subsistence economy, recent archaeological discoveries suggest the existence of settlements, indicating refraining from a “nomadic” interpretation of the communities associated with Yuhuangmiao and related cemeteries, which anyhow rests largely in a misunderstanding of the steppe world where a variety of subsistence practices and a variety of seasonal movements depending on the ecology were practiced. The existence of settlements points to groups that were settled (at least parts of the society). The few isotope analyses undertaken for some individuals buried at Yuhuangmiao point to an agro-pastoral subsistence economy, though we do not know how widespread this was within the community and how that may have changed over time.

Despite the local specifics in the burial practices as well as in the artifacts of Yuhuangmiao, one cannot overlook the general similarities Yuhuangmiao and related cemeteries share with other local phenomena in the northern zone that Rawson has named the Arc. Although we may recognize a few “foreigners”, such as in the special group of elite burials at YHM, for most parts, we see local processes of groups adopting artifacts and rituals and incorporating those in their own practices and rituals. But we also have to bear in mind that archaeologically we only see foreigners if they display their origins visibly in the mortuary arena. The numerous local phenomena within northern China testify to the high degree of connectivity with each other and the neighboring areas to the north and the south and show the intensive engagement with the impulses from the outside. The means of contact are multifold and may range from alliance-building through intermarriage, gift exchange, and trade and can only rarely be identified. This paper highlights the internal dynamics in one very specific area with foreigners from the steppe in the intermediate zone between the Central Plains and the steppes.

## Supplementary Information

Below is the link to the electronic supplementary material.

Supplementary file1 (XLSX 48 KB)

Supplementary file2 (XLSX 392 KB)

Supplementary file3 : **Online Resource 3** 14C dates from the three Yuhuangmiao culture sites produced by two laboratories (BK: Peking University; ZK: IA CASS). Two dates are obtained from charcoal samples. Others are from human or animal bones (Beijingshi 2010, p. 788). Calibration: OxCal v.4.4.4 (Bronk Ramsey 2021) with IntCal20 (Reimer et al. 2020).

High resolution image (TIF 9410 KB)

Supplementary file4 : **Online Resource 4** Initial seriation result. Objects and graves which are removed for various reasons are highlighted.

High resolution image (TIF 2129 KB)

Supplementary file5 : **Online Resource 5** Initial CA result. Objects with outstanding CA plots are highlighted.

High Resolution image (TIF 5030 KB)

Supplementary file6 : **Online Resource 6** Seriation of the HLG and XLG graves. The chronology is determined by comparing the same types of objects from these two sites and those from YHM. The ground plans illustrate graves at HLG and XLG.

High Resolution image (TIF 3808 KB)

Supplementary file7 : **Online Resource 7** Surveyed sites around the Yanhuai Basin (data: Online Resource 1)

High Resolution image (TIF 2942 KB)

Supplementary file8 : **Online Resource 8** The available stable isotope data (Wei 2004) suggests that the carbon and nitrogen levels (related to cereal and meat intake) of individuals in the normal-sized graves remained almost the same level. Isotope data associated with individuals in the large graves is currently unavailable.

High Resolution image (TIF 5030 KB)

Supplementary file 9 (XLSX 48 KB)

Supplementary file10 : **Online Resource 10** Comparison of stable isotope (carbon, nitrogen) results from sites in different regions. Plots show the mean values of adult human samples. Shaded confidence intervals show the isotope levels by region. Three groups between the Mongolian Plateau and the North China Plain: A: the Yan Mountains, northern Taihang Mountains, and Yin Mountains; B: the Yellow River Basin, Fen River Basin, and the eastern Loess Plateau; C: the northern Wei River Basin and the western Loess Plateau (data: Online Resource 9).

High Resolution image (TIF 2942 KB)

## Data Availability

The data used in this article is published data and accessible through the publications cited.

## Footnotes

1 We use the term “Central Plains” to refer to the region covering approximately the eastern low-altitude alluvial plains. The modern name of the main part of the region is the North China Plain, bordered by the Yan Mountains to the north, the Taihang Mountains to the west, the Yangtze River Valley to the south, and the eastern coast.

2 There are several types of decorations that can be attached to clothes or belts. A “slider” is one with a horizontal hollow space and can be attached from left to right. There are also some decorations with a vertical hollow space. In this paper, we separate two types: “plaques” for those relatively large ornaments and “sliders/decorations” for those relatively small objects with a hollow space.

3 The biological sexes of individuals from the three excavated sites are identified based on skeletal morphology, done by Pan Qifeng from the Department of Biological Anthropology, IA CASS (Beijingshi 2010, pp. 675–760).

4 For graves that are not rectangular, the volume is estimated by max length x max width x max depth.

5 A different suggestion by Rawson is that the bronze vessels came from hoards left by the Zhou people and Yuhuangmiao people considered these objects as potential secondary metal resources (Rawson 2023, p. 193).

## Code availability

Tool for Online Seriation and Correspondence Analysis (TOSCA) is used for the seriation and is accessible via the link cited. Radiocarbon dates are calibrated using OxCal v.4.4.4, ©Christopher Bronk Ramsey (Bronk Ramsey 2021). The calibration curve for the northern hemisphere IntCal20 is used (Reimer et al. 2020). Figures are created using Adobe Illustrator, QGIS, and Blender.
