# Supplementary figures and images for: Yuhuangmiao: the socio-cultural dynamics of a community between the steppes and the Chinese plains
Source: Asian Archaeol. 2024 Dec 9;8(2):229–49. doi: 10.1007/s41826-024-00098-4 (PMC11652592; doi:10.1007/s41826-024-00098-4)

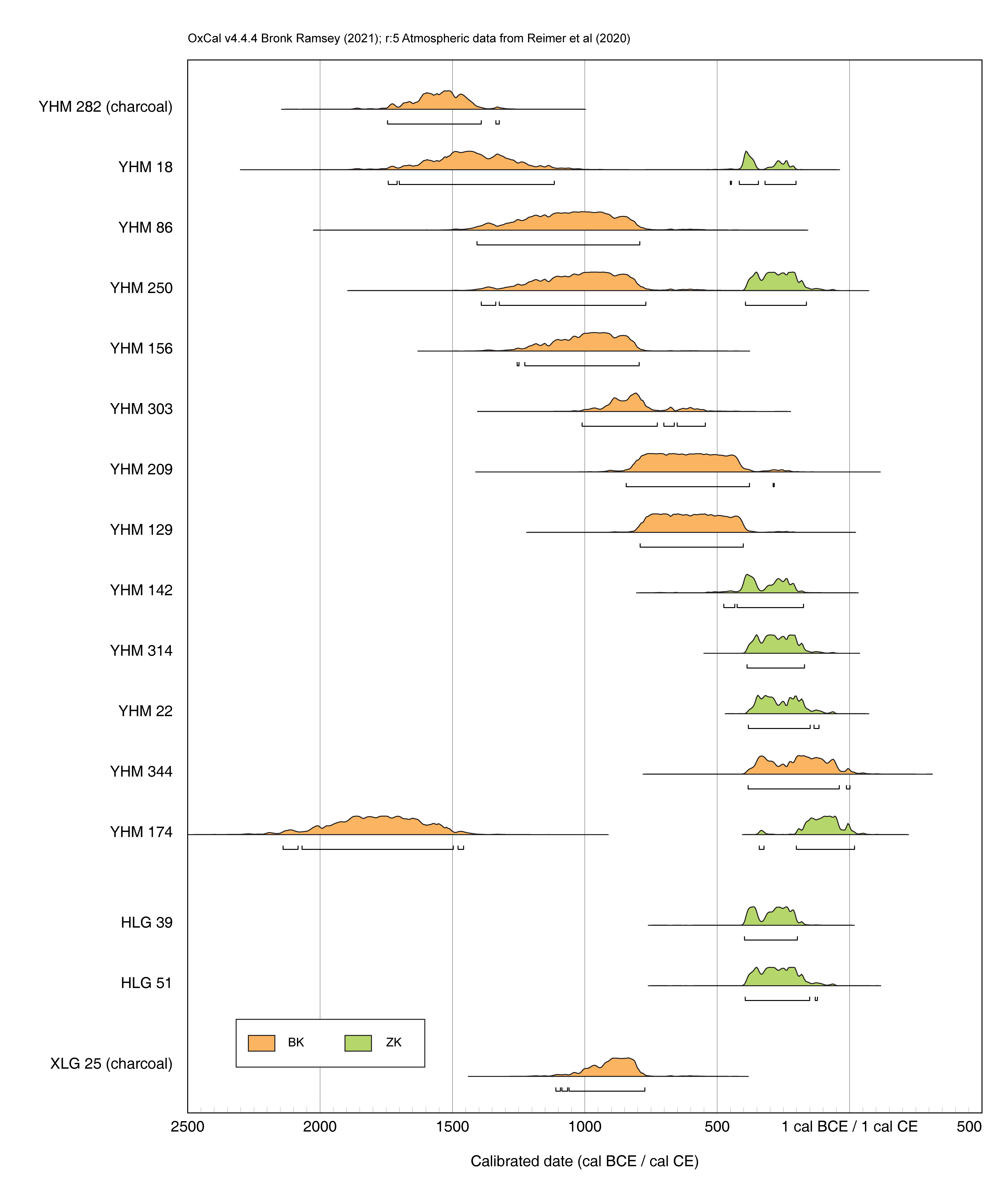

Supplement: Supplementary file 4 — High resolution image (TIF 9410 KB) [file 41826_2024_98_MOESM3_ESM.tif]

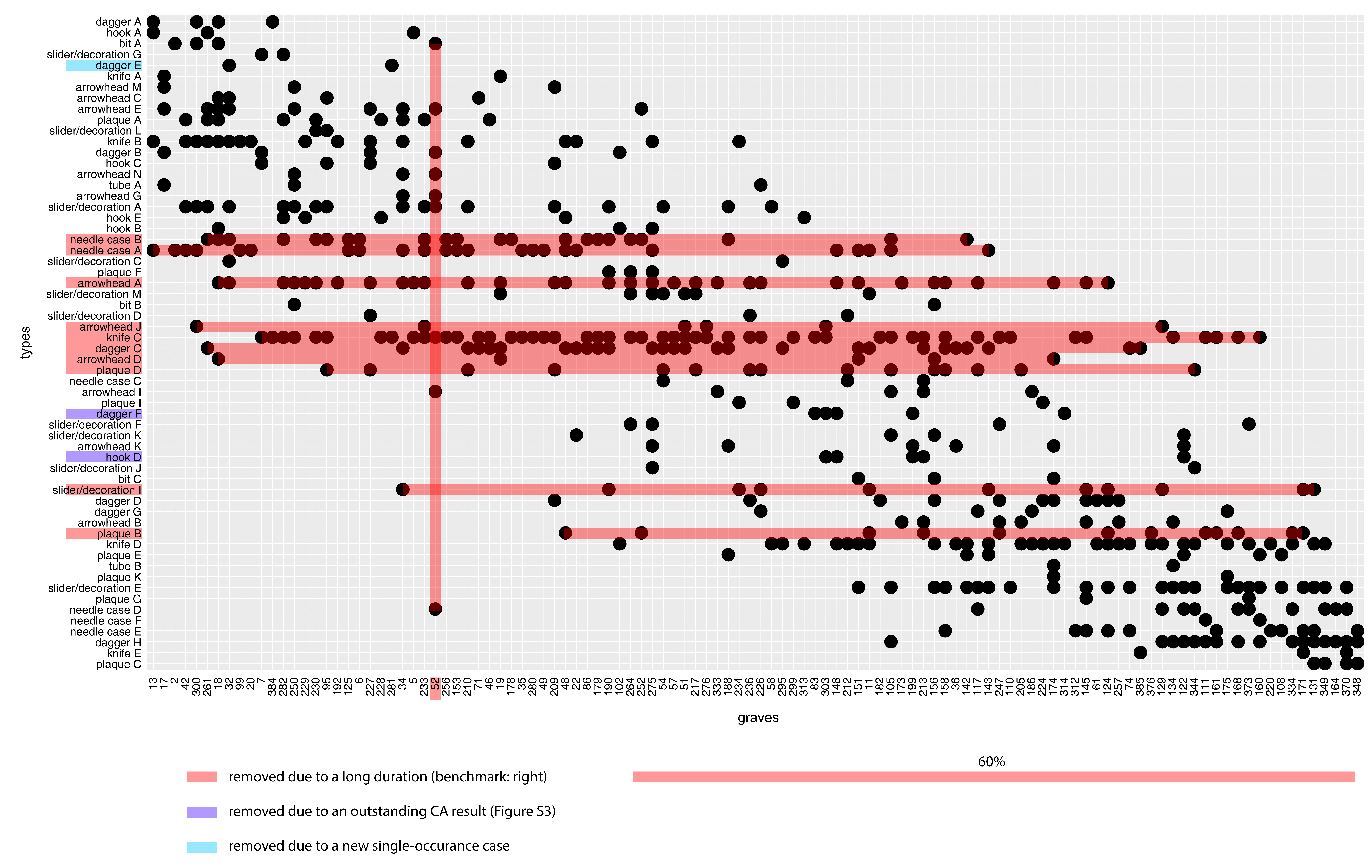

Supplement: Supplementary file 5 — Supplementary file4 : Online Resource 4 Initial seriation result. Objects and graves which are removed for various reasons are highlighted. [file 41826_2024_98_Fig14_ESM.png]

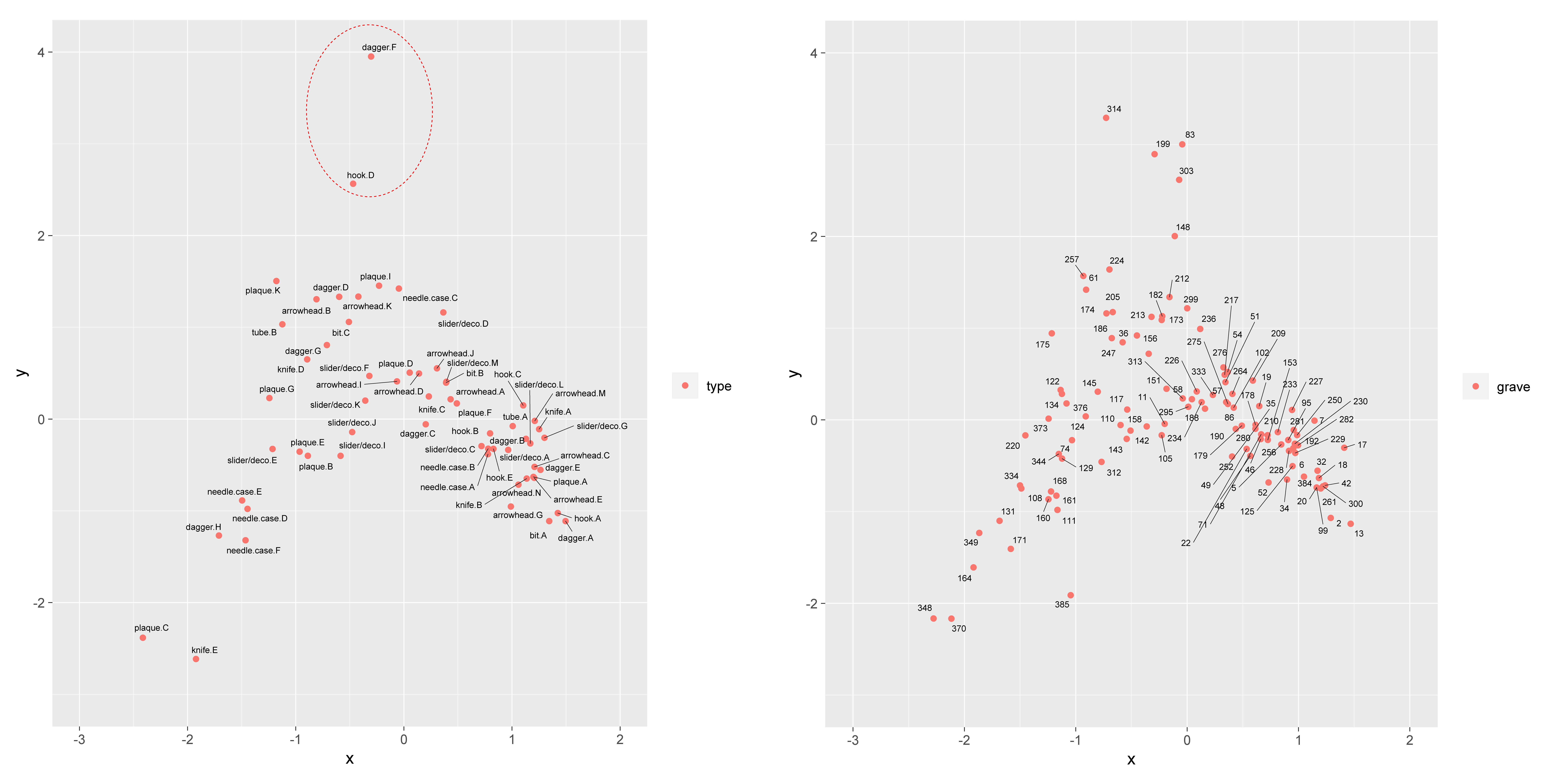

Supplement: Supplementary file 7 — Supplementary file5 : Online Resource 5 Initial CA result. Objects with outstanding CA plots are highlighted. [file 41826_2024_98_Fig15_ESM.png]

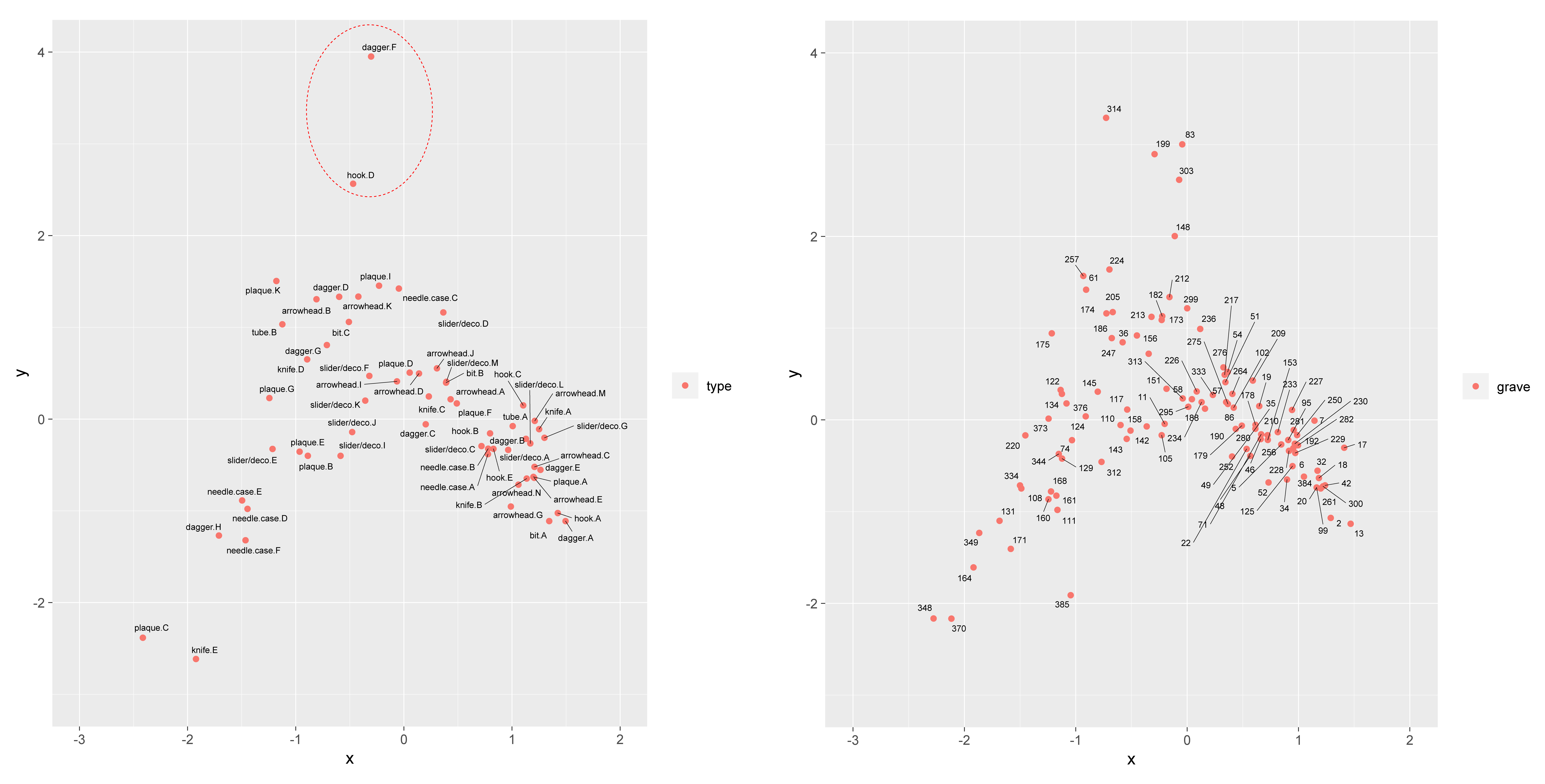

Supplement: Supplementary file 8 — High Resolution image (TIF 5030 KB) [file 41826_2024_98_MOESM5_ESM.tif]

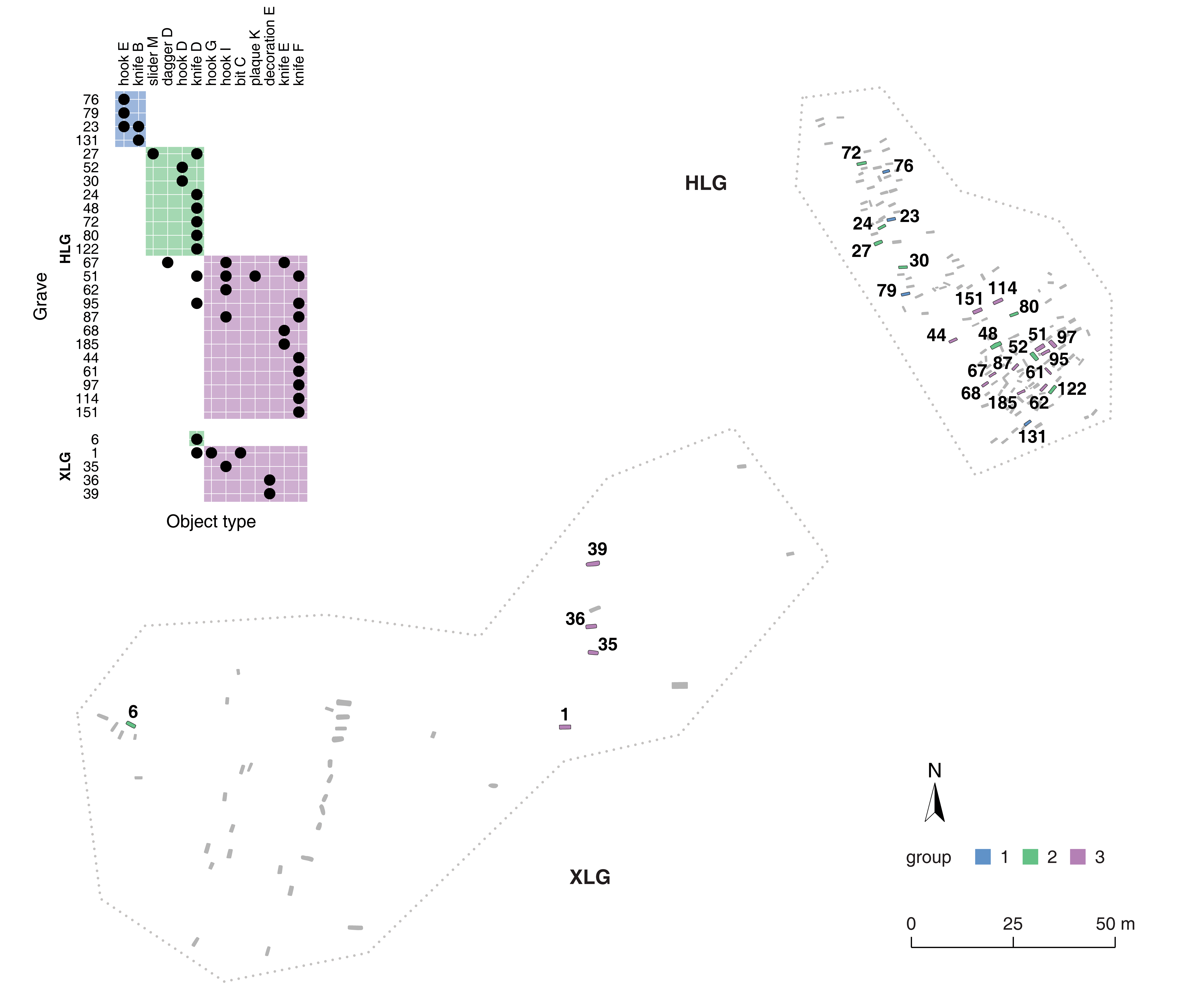

Supplement: Supplementary file 9 — Supplementary file6 : Online Resource 6 Seriation of the HLG and XLG graves. The chronology is determined by comparing the same types of objects from these two sites and those from YHM. The ground plans illustrate graves at HLG and XLG. [file 41826_2024_98_Fig16_ESM.png]

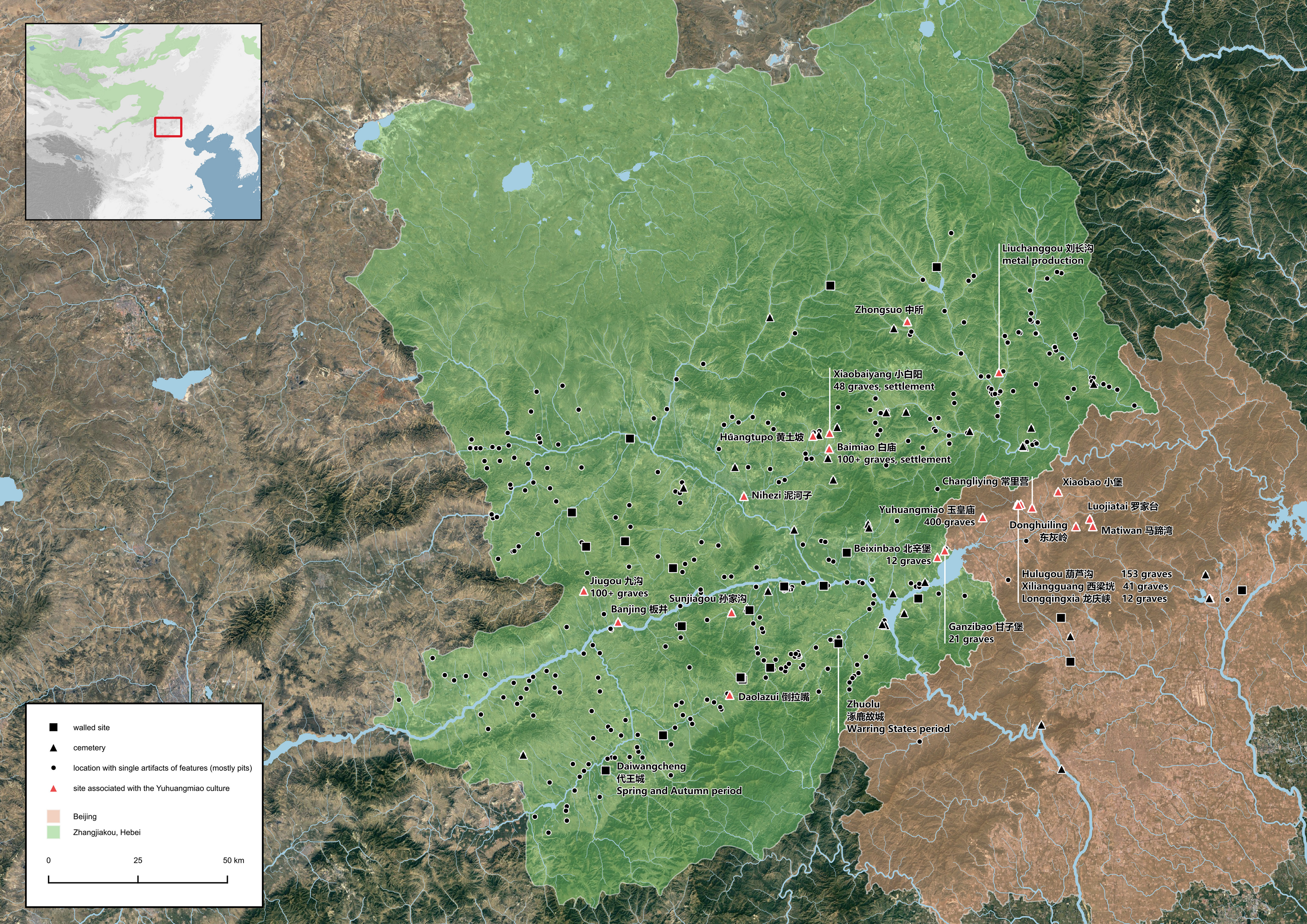

Supplement: Supplementary file 11 — Supplementary file7 : Online Resource 7 Surveyed sites around the Yanhuai Basin (data: Online Resource 1) [file 41826_2024_98_Fig17_ESM.png]

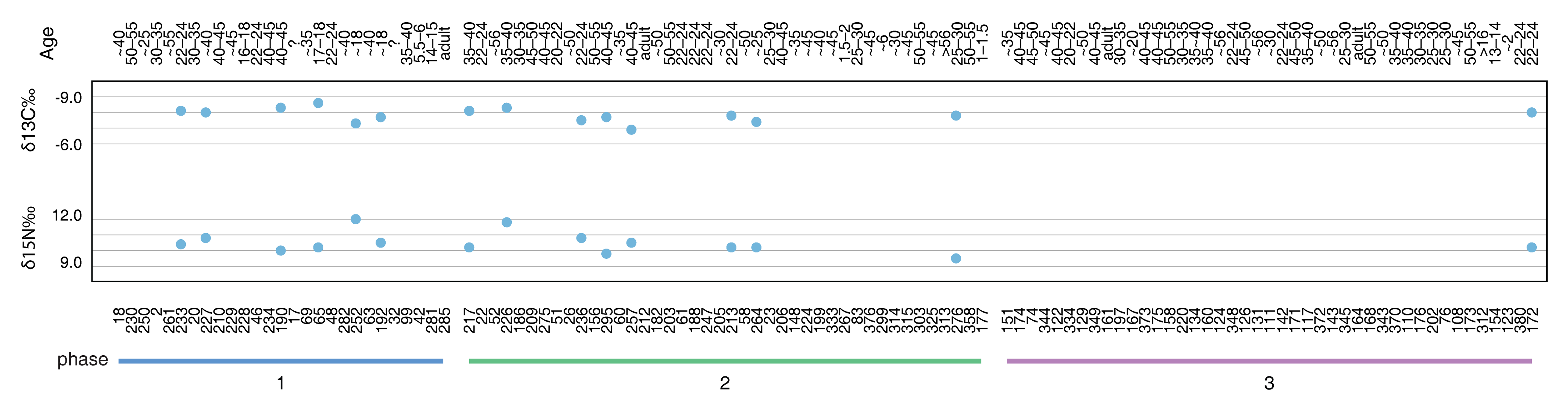

Supplement: Supplementary file 13 — Supplementary file8 : Online Resource 8 The available stable isotope data (Wei 2004) suggests that the carbon and nitrogen levels (related to cereal and meat intake) of individuals in the normal-sized graves remained almost the same level. Isotope data associated with individuals in the large graves is currently unavailable. [file 41826_2024_98_Fig18_ESM.png]

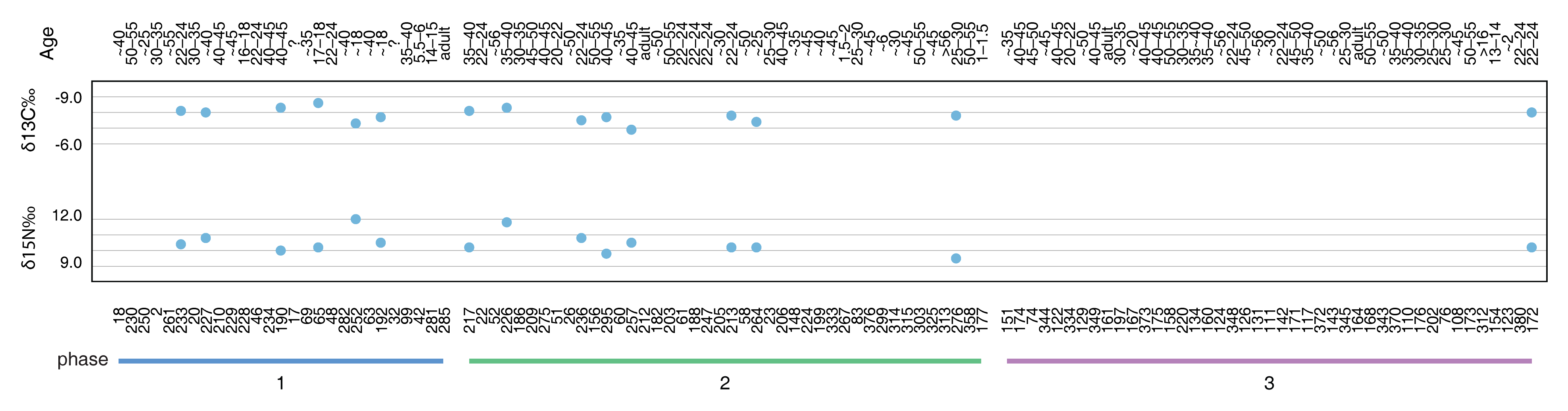

Supplement: Supplementary file 14 — High Resolution image (TIF 5030 KB) [file 41826_2024_98_MOESM8_ESM.tif]

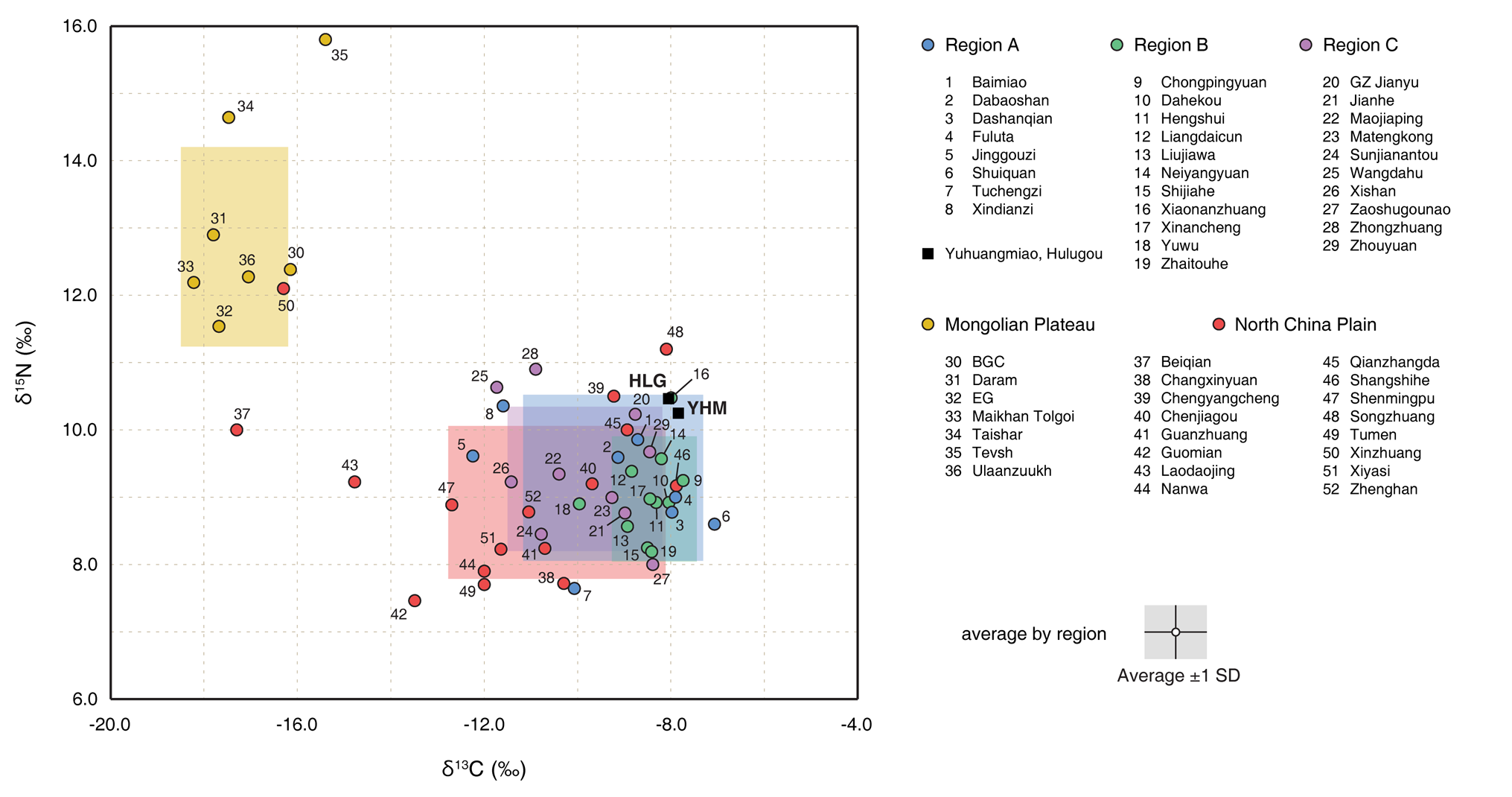

Supplement: Supplementary file 16 — Supplementary file10 : Online Resource 10 Comparison of stable isotope (carbon, nitrogen) results from sites in different regions. Plots show the mean values of adult human samples. Shaded confidence intervals show the isotope levels by region. Three groups between the Mongolian Plateau and the North China Plain: A: the Yan Mountains, northern Taihang Mountains, and Yin Mountains; B: the Yellow River Basin, Fen River Basin, and the eastern Loess Plateau; C: the northern Wei River Basin and the western Loess Plateau (data: Online Resource 9). [file 41826_2024_98_Fig19_ESM.png]

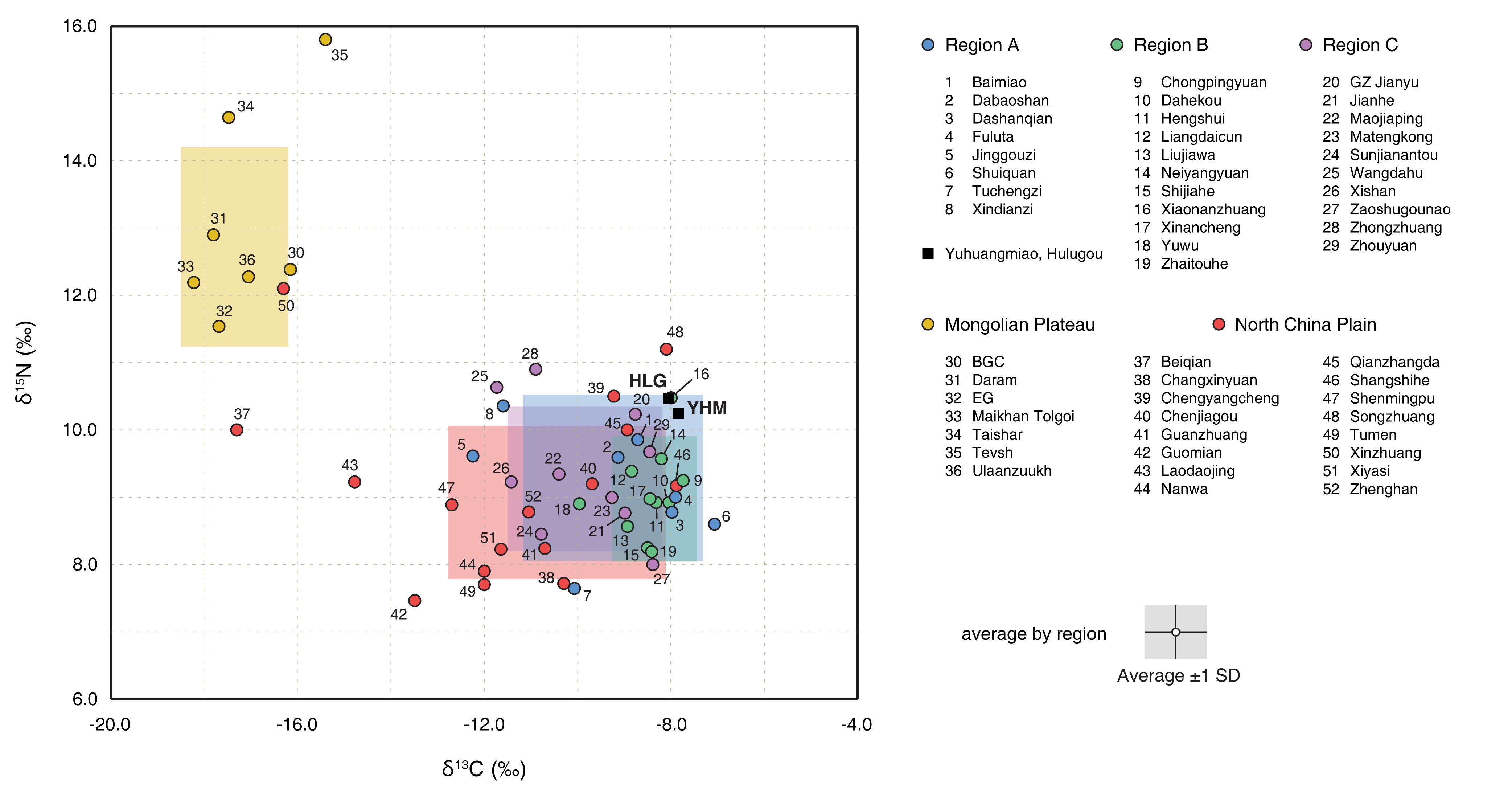

Supplement: Supplementary file 17 — High Resolution image (TIF 2942 KB) [file 41826_2024_98_MOESM10_ESM.tif]
